# A simple and efficient strategy for cell‐based and cell‐free‐based therapies in acute liver failure: hUCMSCs bioartificial liver

**DOI:** 10.1002/btm2.10552

**Published:** 2023-06-02

**Authors:** Lei Feng, Yi Wang, Yu Fu, Adilijiang Yimamu, Zeyi Guo, Chenjie Zhou, Shao Li, Linya Zhang, Jiasheng Qin, Shusong Liu, Xiaoping Xu, Zesheng Jiang, Shaoru Cai, Jianmin Zhang, Yang Li, Qing Peng, Xiao Yi, Guolin He, Ting Li, Yi Gao

**Affiliations:** ^1^ Department of Hepatobiliary Surgery II, Zhujiang Hospital Southern Medical University Guangzhou Guangdong China; ^2^ Guangdong Provincial Research Center for Artificial Organ and Tissue Engineering, Guangzhou Clinical Research and Transformation Center for Artificial Liver, Institute of Regenerative Medicine, Zhujiang Hospital Southern Medical University Guangzhou Guangdong China; ^3^ State Key Laboratory of Organ Failure Research Southern Medical University Guangzhou Guangdong China

**Keywords:** acute liver failure, bioartificial liver, cell apoptosis, hUCMSCs, small extracellular vesicles

## Abstract

Acute liver failure (ALF) is a life‐threatening condition. Cell‐based and cell‐free‐based therapies have proven to be effective in treating ALF; however, their clinical application is limited by cell tumorigenicity and extracellular vesicle (EV) isolation in large doses. Here, we explored the effectiveness and mechanism of umbilical cord mesenchymal stem cells (hUCMSCs)‐based bioartificial liver (hUCMSC‐BAL), which is a simple and efficient strategy for ALF. D‐galactosamine‐based pig and mouse ALF models were used to explore the effectiveness of hUCMSC‐BAL and hUCMSC‐sEV therapies. Furthermore, high‐throughput sequencing, miRNA transcriptome analysis, and western blot were performed to clarify whether the miR‐139‐5p/PDE4D axis plays a critical role in the ALF model in vivo and in vitro. hUCMSC‐BAL significantly reduced inflammatory responses and cell apoptosis. hUCMSC‐sEV significantly improved liver function in ALF mice and enhanced the regeneration of liver cells. Furthermore, hUCMSC‐sEV miRNA transcriptome analysis showed that miR‐139‐5p had the highest expression and that PDE4D was one of its main target genes. The sEV miR‐139‐5p/PDE4D axis played a role in the treatment of ALF by inhibiting cell apoptosis. Our data indicate that hUCMSC‐BAL can inhibit cytokine storms and cell apoptosis through the sEV miR‐139‐5p/PDE4D axis. Therefore, we propose hUCMSC‐BAL as a therapeutic strategy for patients with early ALF.

## 1 INTRODUCTION

Acute liver failure (ALF) is a rare, heterogeneous liver dysfunction with high incidence and mortality rates, characterized by jaundice, coagulation disorders, hepatorenal syndrome, and hepatic encephalopathy (HE), and develops in the absence of previous acute liver disease.^1^ The pathological mechanism of early ALF includes a cytokine storm, homeostasis, and immune environment imbalance, leading to substantial hepatocyte death.^2^, ^3^, ^4^, ^5^ The release of metabolites, inflammatory mediators, and toxic metabolites caused by ALF and the ability of residual liver cells to repair liver function determine the prognosis of ALF. That is, hepatocyte injury is attributed to inflammatory mediators and toxic metabolites released by early ALF, which results in an imbalance of homeostasis and the immune environment of surrounding normal hepatocytes.^1^ This causes the death of surrounding normal hepatocytes and produces a cascade reaction, resulting in a large number of hepatocyte deaths and irreversible liver injury. Therefore, controlling the inflammatory response and homeostasis may be a mechanism of treating early ALF.

At present, liver transplantation is the only effective treatment for ALF patients; however, its application is limited by a shortage of liver donors, complex surgery, high costs, and the need for lifelong immunosuppressant use.^6^ Artificial livers are a key area of research in ALF treatment that have emerged in recent years, which include non‐bioartificial, bioartificial, and hybrid artificial livers. Although non‐bioartificial livers are widely used in clinical applications, they cannot significantly improve the survival time of patients with ALF, except with high plasma exchange.^7^ Conversely, bioartificial and combined artificial livers are limited by their seed cells and bioreactors, and most of them are considered to still be in the preclinical stage, and thus have not been applied in clinical settings except the bioartificial liver of HepArt Medical Devices (AMC‐BAL) and Vital Therapies (ELAD).^8^ However, large clinical trials with AMC‐BAL^9^ and ELAD^10^ did not show a survival benefit. Although scientists have extensively studied seed cells from various sources and their corresponding bioreactors,^11^, ^12^, ^13^, ^14^, ^15^, ^16^, ^17^, ^18^ most previous literature focused on liver cell function replacement therapy and are limited by the lack of human liver cell sources and the tumorigenicity of liver tumor cell lines, as well as safety and ethical concerns. Nevertheless, bioartificial livers that act solely on the cytokine storm, homeostasis regulation, and immune regulation have not previously been reported.

Mesenchymal stem cell‐based and cell‐free‐based therapies have proven to be effective in treating ALF. Recently, studies have shown that mesenchymal stem cell (MSC) transplantation can rescue ALF through the differentiation and/or secretion of growth factors, cytokines, and chemokines.^19^, ^20^, ^21^, ^22^, ^23^, ^24^ However, MSC transplantation, typically local or systemic intravenous injection of MSCs, may cause the release of inflammatory factors, an enhanced risk of opportunistic infections and cancer, allogeneic production of antibodies, and other adverse consequences.^25^ In contrast, veinous infusion of MSCs may cause blood vessel clots. Interestingly, an increasing number of studies have shown that extracellular vesicles (EV), mainly small EV (sEV, derived from MSCs regulate immunity and inflammatory response and promote cell proliferation.^25^ However, although sEV derived from MSCs show broad therapeutic prospects, their clinical application is limited by factors, such as the difficulty of large‐scale isolation and purification and low yield.^26^, ^27^, ^28^

To address these questions and demonstrate the value of human umbilical cord mesenchymal stem cells (hUCMSCs) for regenerative medicine in ALF, we first constructed a bioartificial liver based on hUCMSCs (hUCMSC‐BAL), which we then verified using a pig ALF model. Not only could hUCMSC‐BAL avoid adverse reactions caused by cells entering the patient's body, but also sEV and cytokines secreted by hUCMSCs could enter the patient's body through the bioreactor semi‐permeable membrane to play a therapeutic role. To further explore the specific mechanism of hUCMSC‐BAL, we extracted sEV and factors in the circulating culture medium of the hUCMSCs bioreactor and constructed a mouse ALF model using sEV from the hUCMSCs bioreactor for treatment. sEV transcriptome analysis revealed that miR‐139‐5p was the most highly expressed miRNA and that PDE4D is one of its main target genes. These results were verified in L02 and HepaRG cell ALF model.

## 2 RESULTS

### 2.1 hUCMSCs express MSC‐specific markers and are capable of multilineage differentiation

**FIGURE 1:**
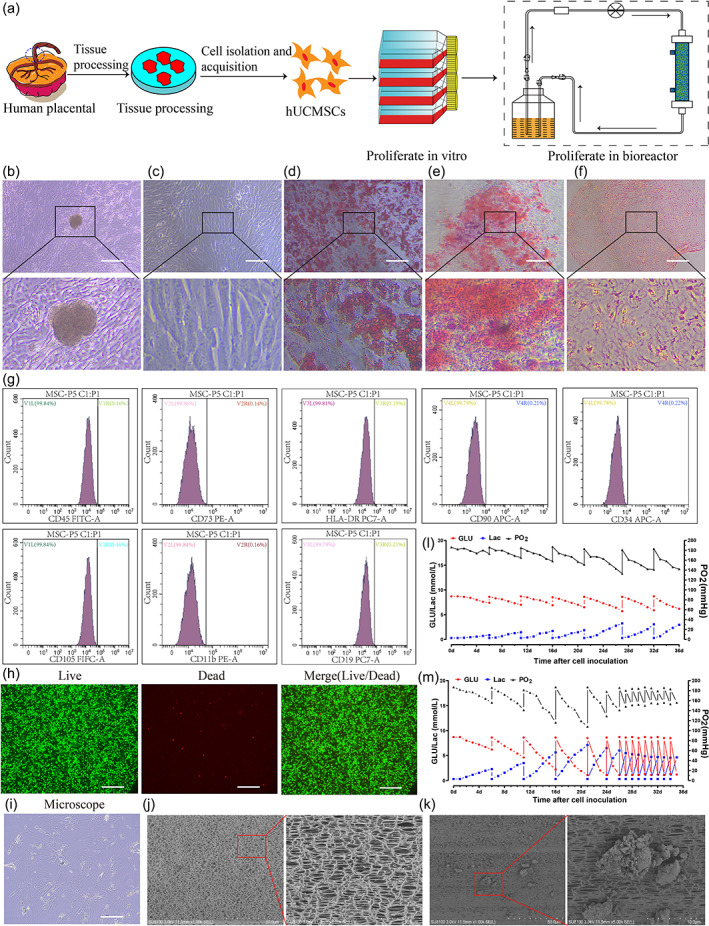
hUCMSCs acquisition and identification, and large‐scale expansion in hollow fiber bioreactor. (a) Schematic diagram of acquisition and identification, and large‐scale expansion of hUCMSCs, which were first expanded to 10^7^–10^8^ in the culture flask, then inoculated into the hollow fiber bioreactor until amplified to 10^9^–10^10^; (b) hUCMSCs were isolated by the tissue block method; (c) P5‐generation hUCMSCs morphology under an optical inverted microscope. (d) hUCMSCs differentiated into fat and stained with Oil red O (×200). (e) Osteogenic differentiation of hUCMSCs stained with alizarin red (×200). (f) Cartilage differentiation of hUCMSCs stained with safranin O (×200). (g) Flow cytometry showed that hUCMSCs highly expressed CD73, CD90, and CD105 (both ≥ 95%) but did not express CD34, CD45, CD19, CD11b, or HLA‐DR (both ≤ 5%); (h) The results of live and dead staining of hUCMSCs in the bioreactor; (i) hUCMSCs in the bioreactor under a light microscope; (j) Hollow‐fiber superfine structure in the bioreactor. (k) Adhesion of hUCMSCs to hollow fiber in the bioreactor. (l–m) Changes in GLU, Lac, and PO_2_ in the bioreactor during hUCMSCs culture in (l) Group B and (m) Group C. GLU, glucose; Lac, lactate; PO_2_, oxygen partial pressure. Scale bar 200 μm.

The cultured hUCMSCs showed a uniform, slender, spindle shape and a typical spiral arrangement under the microscope (Figure 1c). Many cells were observed to exhibit a division pattern, and they grew vigorously. The flow cytometry results of hUCMSCs showed that the cells expressed the MSC surface markers CD73 (98.4%), CD90 (99.4%), and CD105 (99.8%) (positive rate ≥ 95%) but did not express the hematopoietic cell markers CD34 (0.22%), CD45 (0.16%), CD19(0.21%), CD11b (0.16%), or HLA‐DR (0.19%) (positive rates ≤ 5%) (Figure 1g). These data showed that hUCMSCs are homogeneous, without hematopoietic or endothelial cells, and have good MSC characteristics. After 21 days of culture in osteogenic, adipogenic, and chondrogenic induction media, alizarin red, oil red O, and safranin O staining was positive (Figure 1d–f). The above experimental results confirmed that hUCMSCs are capable of multilineage differentiation.

### 2.2 Large‐scale expansion of hUCMSCs in the bioreactor

At present, an important challenge for bioartificial liver application is the large‐scale production of seed cells (up to 10^9^–10^10^ cells). Therefore, we constructed an hUCMSCs bioreactor culture system (fiber surface area 4000 cm^2^/1.2 m^2^, fiber outer cavity volume 20 mL/70 mL). hUCMSCs were implanted in the outer cavity of the hollow fiber of the bioreactor and attached to the surface of the hollow fiber (Figure 1h–k). The serum‐free medium passed through the inner cavity of the hollow fiber using a peristaltic pump and exchanged substances through the semi‐permeable membrane. sEV, secretory proteins, and other molecules could freely pass through the hollow fiber semi‐permeable membrane, whereas cells and cell fragments could not. We determined the proliferation rate of cells based on the rate of glucose (GLU) decline and lactate increase in the culture medium (Figure 1 l,m). Because the rate of GLU decline is directly proportional to the number of cells in the bioreactor, we considered the expansion of hUCMSCs in the bioreactor to be complete when the rate of GLU decline was stable for 1 week.

### 2.3 Establishment of the porcine ALF model

To verify the efficacy of hUCMSC‐BAL, we induced an ALF model in miniature pigs by intravenous injection of D‐gal at 0.40 g/kg body weight according to our previous study,^4^ thereby establishing an animal model with similar characteristics to human ALF. The blood alanine transaminase (ALT), aspartate transaminase (AST), total bilirubin (TBIL), ammonia (Amm) levels, prothrombin time (PT), and the international normalized ratio (INR) in experimental animals increased significantly (*p*s < 0.05) after D‐gal injection, meeting the diagnostic criteria of ALF.^4^ All ALF model pigs showed clinical ALF symptoms, such as anorexia, unstable gait, and yellow urine. These results confirm the successful construction of the ALF animal model.

### 2.4 Prevention of ALF progression by 8‐h hUCMSC‐BAL treatment

**FIGURE 2:**
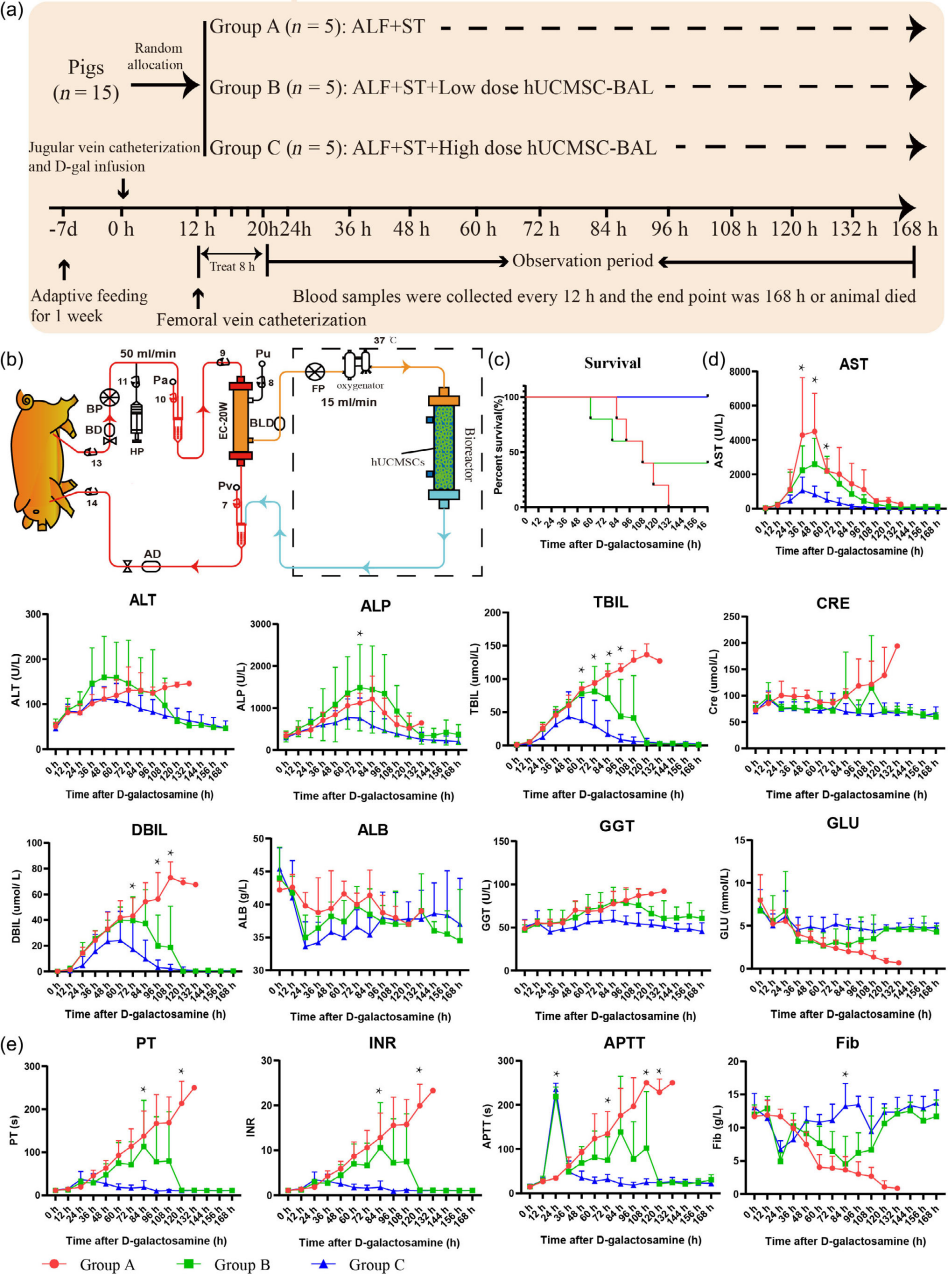
hUCMSC‐BAL treatment could relieve porcine ALF liver injury and coagulation dysfunction and prolong survival time. (a) Experimental design, observation and treatment timeline, ALF, acute liver failure; ST, standard treatment; BAL, bioartificial liver; D‐Gal, D‐galactosamine. (b) Schematic diagram of hUCMSC‐BAL; AD, bubble detector; BD, blood supply deficiency detector; BLD, blood leakage detector; BP, blood pump; EC‐20 W, plasma separator; FP, points slurry pump; HP, heparin pump; Pa, prefilter pressure; Pu, filter pressure; Pv, post‐filter pressure. (c) Kaplan–Meier survival curve (*n* = 5 for each group) for the three groups, log‐rank test. (d) Dynamic changes in liver and renal function indexes, that is, plasma AST, ALT, DBIL, TBIL, ALP, ALB, GGT, CRE, and GLU (*n* = 5 for each group). (e) Changes in coagulation function parameters of the three groups, that is, PT, INR, APTT, and Fib (*n* = 5 for each group). Group A: ALF + ST; Group B: ALF + ST + low dose hUCMSC‐BAL; Group C: ALF + ST + high dose hUCMSC‐BAL. Mean ± SD, *p < 0.05 using unpaired Student's *t*‐test or one‐way ANOVA.

Survival analysis showed that the survival rate of group C (5/5) was better than that of groups B (3/5) and A (0/5) (Figure 2c). All animals showed anorexia and activity reduction, their liver function‐related indicators (Amm, ALT, AST, alkaline phosphatase (ALP), TBIL, and direct bilirubin (DBIL)) began to increase, blood GLU began to decline, and coagulation function (PT, activated partial thromboplastin time (APTT), and fibrinogen (Fib) began to deteriorate after 12 h of D‐gal administration, but there was no significant difference between groups. Biochemical measurements showed that high‐dose hUCMSC‐BAL treatment reduced Amm concentrations at 24 h after D‐gal administration compared with those in the ALF control and low‐dose hUCMSC‐BAL groups (Figure 2d). Pigs in group A showed obvious symptoms of HE 48 h after D‐gal administration. However, none of the animals in group C presented with the characteristic symptoms of HE. Liver function (AST, ALT, TBIL, ALP, gamma‐glutamyl transferase (GGT), and DBIL) in group C gradually recovered after hUCMSC‐BAL treatment compared with that in groups A and B. While GLU continuously declined in groups A and B, ALB in groups B and C declined after hUCMSC‐BAL treatment, then gradually recovered (Figure 2d). There were no significant differences in creatinine (CRE) levels among the three groups, except for a significant increase before death (Figure 2d). For coagulation function, PT, INR, and APTT increased at 12 h after D‐gal administration, whereas Fib began to decrease. Because of heparinization during hUCMSC‐BAL treatment, APTT and PT increased significantly, and Fib decreased significantly at 24 h, but recovered at 36 h in groups B and C. The coagulation function (PT, INR, APTT, and Fib) of the surviving animals in groups B and C reached a peak, and then returned to baseline, whereas the coagulation function of dead animals in groups A and B continued to worsen (Figure 2e). Overall, hUCMSC‐BAL treatment reduced blood Amm, improved liver function and coagulation function, and stabilized the homeostasis environment in the D‐gal‐induced ALF porcine model.

To evaluate the safety and efficacy of hUCMSC‐BAL during treatment, we collected samples to detect biochemical indicators every 2 h during treatment and monitored the general condition, heart rate, respiratory rate, blood pressure, and oxygen saturation of the experimental animals. hUCMSC‐BAL was stable, and no obvious abnormality was observed during treatment. There were no significant differences in GGT, GLU, ALB, BUN, CRE, or APTT between the two groups during hUCMSC‐BAL treatment; however, significant differences were observed in Amm, ALT, AST, ALP, TBIL, and DBIL (Figure S2a–d). Vital signs remained stable during 8 h of treatment, except for the systolic blood pressure (SP) and diastolic blood pressure (DP), which decreased slightly after anesthesia, and no significant difference was observed between two groups (Figure S2e). The prefilter pressure, post‐filter pressure, and transmembrane pressure of the plasma separator remained stable throughout hUCMSC‐BAL treatment (Figure S2f). Cell death/live staining showed that cell viability was more than 90% before and after hUCMSC‐BAL treatment (Figure S2g).

### 2.5 hUCMSC‐BAL therapy alleviated peripheral inflammation

**FIGURE 3:**
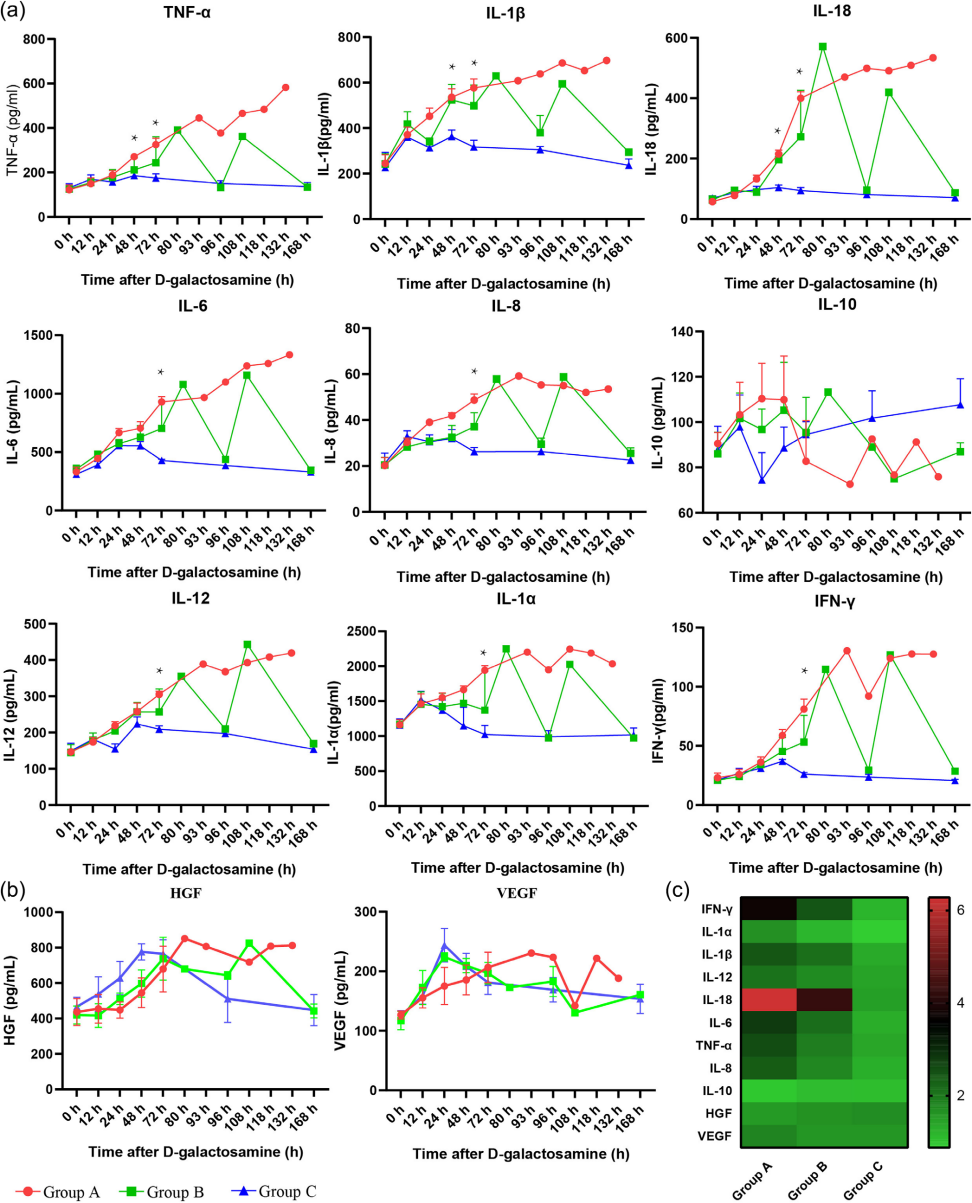
hUCMSC‐BAL treatment could reduce porcine ALF inflammatory reaction. (a) Inflammatory parameters, including TNF‐α, IL‐1β, IL‐18, IL‐6, IL‐8, IL‐10, IL‐12, IL‐1α, and INF‐γ (*n* = 4 per group). (b) HGF and VEGF concentrations in each group (*n* = 4 per group). (c) A heatmap of inflammatory cytokines for the three groups at 72 h. Group A: ALF + ST; Group B: ALF + ST + low dose hUCMSC‐BAL; Group C: ALF + ST + high dose hUCMSC‐BAL.*p < 0.05 using unpaired Student's *t*‐test or one‐way ANOVA.

ALF animals exhibit features of systemic inflammation, progression to multiple organ dysfunction, and immune imbalance. In this study, all inflammatory cytokines were significantly altered in the early stage of ALF, suggesting that drug‐induced ALF is closely related to the inflammatory cascade. The levels of pro‐inflammatory cytokines (IFN‐γ, TNF‐α, IL‐1α, IL‐1β, IL‐6, IL‐8, IL‐12, and IL‐18) in group A significantly increased and peaked before animal death. Most pro‐inflammatory cytokines in group C decreased slightly after hUCMSC‐BAL treatment, which may be related to hemodilution and filtration. Subsequently, the above factors increased slightly again, and then reached a peak value at 48 h after modeling before decreasing, with the values lower than those for groups A and B at the same time. With the recovery of liver function, the above inflammatory cytokines gradually returned to baseline levels in groups B and C (Figure.3a). The levels of anti‐inflammatory cytokines (IL‐10) and growth factors (hepatocyte growth factor (HGF) and vascular endothelial growth factor VEGF) increased after ALF modeling and peaked at 48 h, with no significant difference among the three groups (Figure 3b). Based on the baseline levels of inflammatory cytokines, heat maps at 72 h after modeling showed that pro‐inflammatory factors (IFN‐γ, TNF‐α, IL‐1α, IL‐1β, IL‐6, IL‐8, IL‐12, and IL‐18) increased the most in group A, followed by group B, whereas no significant increase was observed in group C. The increase in IL‐18 was the most obvious, and the difference between the groups was the largest. Anti‐inflammatory cytokines (IL‐10) and related growth factors (HGF and VEGF) did not increase significantly, and there was no significant difference between groups (Figure 3c).

### 2.6 hUCMSC‐BAL therapy alleviates liver injury, enhances liver regeneration, and alleviates HE

**FIGURE 4:**
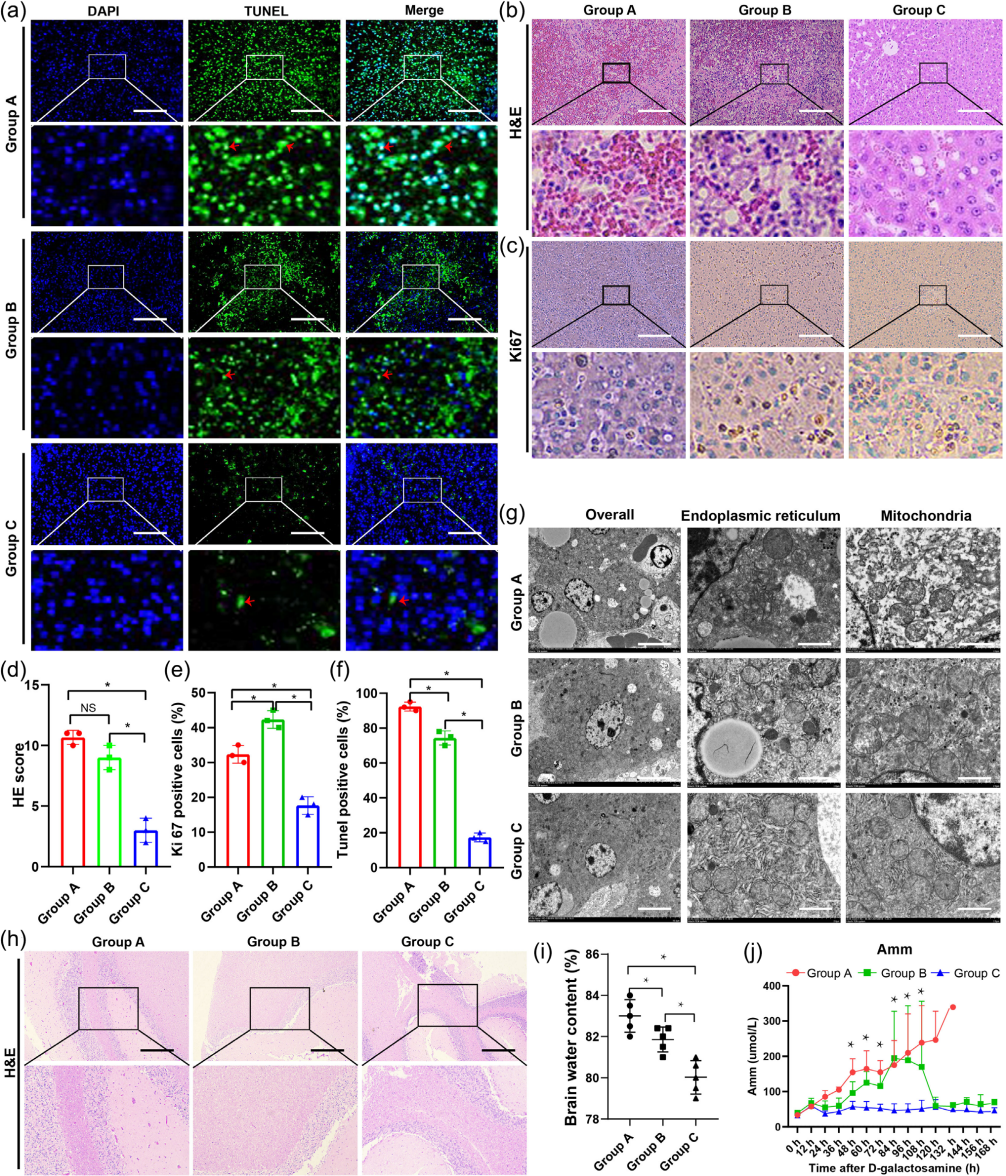
hUCMSC‐BAL treatment could reduce cell apoptosis and HE and promote liver regeneration. (a) TUNEL staining of liver tissue of ALF animals in three groups, scale bar 200 μm. (b) H&E staining of liver tissue of ALF animals in the three groups, scale bar 200 μm, plus illustration at high magnification. (c) Ki67 staining of liver tissue of ALF animals in the three groups, scale bar 200 μm, plus illustration at high magnification. (d) Liver injury score of the three groups; (e) Comparison of Ki67‐positive cells in the three groups; (f) Comparison of TUNEL‐positive rates among the three groups; (g) Electron microscopic of liver tissue of ALF animals in the three groups; scale bar 200 μm. (h) H&E staining of brain tissue of ALF animals in the three groups, scale bar 200 μm, plus illustration at high magnification. (i) Brain water content in the three groups; (j) Dynamic changes in blood ammonia in the three groups (*n* = 5). *p < 0.05 using unpaired Student's *t*‐test or one‐way ANOVA; Group A: ALF + ST; Group B: ALF + ST + low dose hUCMSC‐BAL; Group C: ALF + ST + high dose hUCMSC‐BAL; HE: Hepatic encephalopathy; H&E: Hematoxylin–eosin.

To evaluate the pathological changes in ALF animals, hematoxylin–eosin (H&E), Ki67, and TUNEL staining and electron microscopy were performed after the animals died or after the end point of observation. TUNEL staining and TUNEL‐positive cells showed that hepatocyte apoptosis in group C was lower than that in groups A and B (Figure 4a,f). H&E staining showed extensive hemorrhagic necrosis, disorder of the hepatic lobule structure, and vacuoles in group A, which was more severe than that in groups B and C (Figure 4b). The HE scores in group C were lower than those in groups A and B (Figure 4d). Ki67 staining and Ki67‐positive cells showed that hepatocyte regeneration in group B was higher than that in groups A and C (Figure 4c,e). Electron microscopy showed that endoplasmic reticulum and mitochondrial damage in group C was less than that in groups A and B (Figure 4g). However, Masson, Sirius red, PAS, and Oil red staining in the three groups showed no significant differences (Figure S3). Gross necroscopy specimens and H&E staining of extrahepatic organs in the three groups showed no significant changes (Figure S4). The above results showed that hUCMSC‐BAL can inhibit cell apoptosis, promote liver cell regeneration, and prolong the survival of ALF animals.

HE features cerebral edema and neuronal dysfunction, often induced by hyperammonemia. In addition to directly causing cerebral edema, hyperammonemia also induces neuroinflammation, mediating cognitive impairment. The brain water content was used to evaluate cerebral edema and was the lowest in group C compared with that in the other groups (*p* < 0.05) (Figure 4h,i), and the Amm was lower in group C than in the other groups (*p* < 0.05) (Figure 4j). These findings suggest that hUCMSC‐BAL therapy has a significant protective effect on the brain by alleviating cerebral edema and Amm.

### 2.7 hUCMSC‐sEV express the morphological characteristics and specific markers of sEV

**FIGURE 5:**
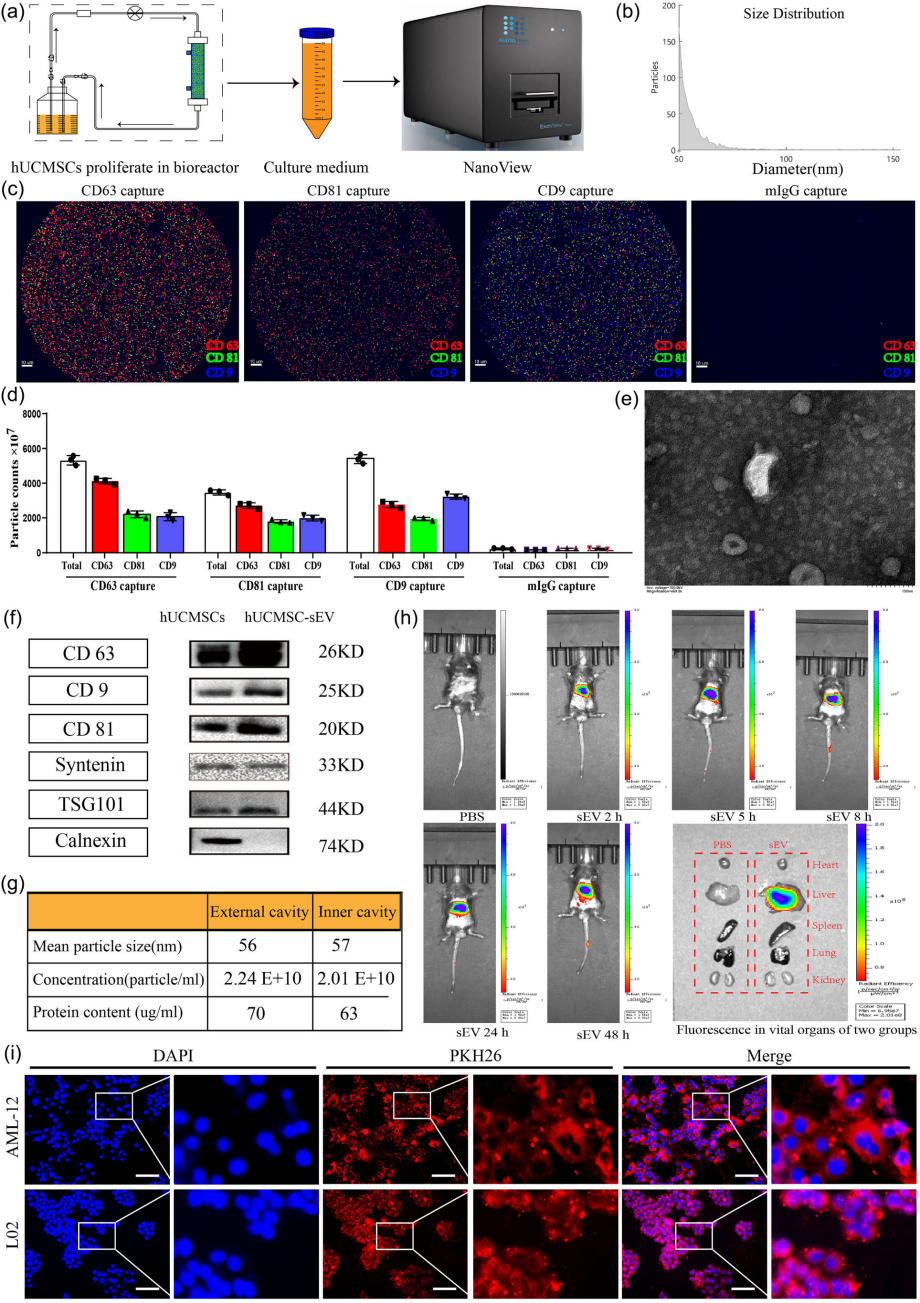
hUCMSC‐sEV identification and tracing in vivo and in vitro. (a) hUCMSC‐sEV acquisition and identification diagram. (b) sEV size according to NanoView. (c) Representative fluorescence images of different capture spots demonstrating colocalization of CD63, CD81, CD9, and mIgG on a single sEV. (d) sEV concentration detection at different capture spots. (e) Results of electron microscopic examination of sEV. (f) Western blotting results of sEV markers. (g) Comparison of particle size, concentration, and protein content of secretions in the internal and external chambers of the bioreactor. (h) In vivo fluorescence imaging tracing of hUCMSC‐sEV. (i) In vitro fluorescence imaging tracing of hUCMSC‐sEV.

To further explore the mechanism of hUCMSC‐BAL in the treatment of ALF, we successfully extracted sEV from the circulating culture medium of the hUCMSCs bioreactor (Figure 5a), and found no significant difference in the protein content of the inner and outer hollow fibers in the bioreactor (Figure 5g). The NanoView results showed that the average particle size of hUCMSC‐sEV was approximately 56 nm (Figure 5b–d). Transmission electron microscopy showed that the sEV were disc‐shaped, with a diameter of approximately 100 nm (Figure 5e). Western blotting results confirmed that hUCMSC‐sEV highly expressed the sEV protein markers CD9, TSG101, CD81, syntenin, and CD63, but did not express the negative protein marker calnexin (Figure 5f). The above experimental results prove that the sEV isolated from the culture supernatant of the hUCMSCs bioreactor have the typical morphology, good uniformity, and characteristics of sEV.

To confirm that hUCMSC‐sEV could be taken up by the liver, we labeled hUCMSC‐sEV with DIR, which is a fluorescent dye that binds to the phospholipid bilayer membrane, and observed them in vivo and in vitro under a fluorescence microscope within 48 h. The results showed that the liver could take up hUCMSC‐sEV after injection through the tail vein (Figure 5h) and that hUCMSC‐sEV could be taken up by L02 and AML‐12 cells (Figure 5i).

### 2.8 hUCMSC‐sEV relieves ALF in mice

**FIGURE 6:**
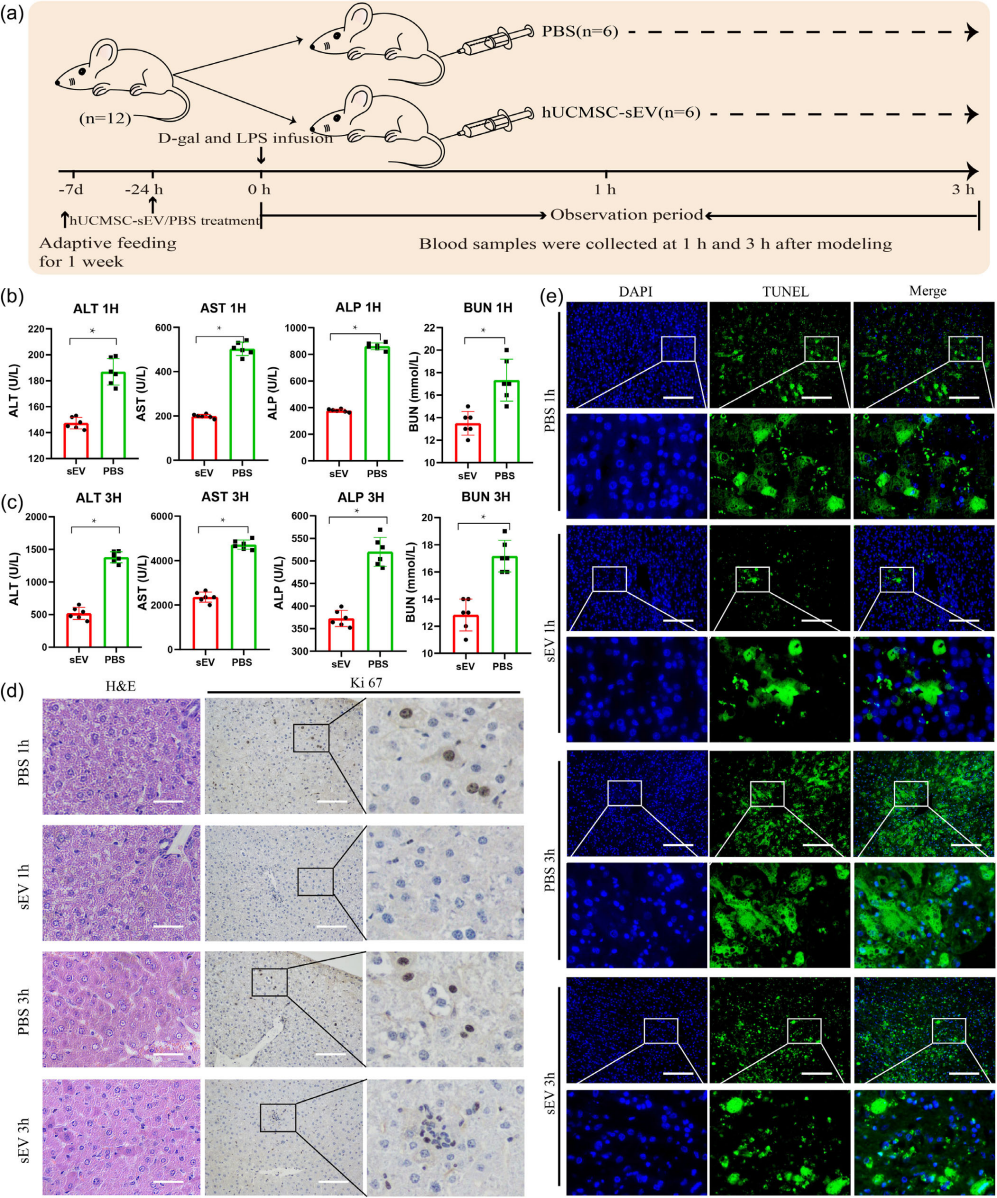
Evaluation of hUCMSC‐sEV treatment in the mouse ALF model. (a) Experimental design timeline. (b) Changes in liver and renal function indexes at 1 h after modeling between the two groups for ALT, AST, ALP, and BUN (*n* = 3 in each group). (c) Changes in liver and renal function indexes at 3 h after modeling between the two groups for ALT, AST, ALP, and BUN (*n* = 3 in each group). (d) H&E and Ki67 staining of liver tissue at 1 and 3 h after modeling between two groups. (e) TUNEL staining of liver tissue at 1 and 3 h after modeling between two groups. H&E: Hematoxylin–eosin; *p < 0.05 by unpaired‐tailed Student's *t*‐test.

To further explore the relevant mechanisms, we successfully constructed a mouse ALF model using D‐gal and LPS and treated mice with hUCMSC‐sEV (Figure 6a). Compared with control, hUCMSC‐sEV injection significantly alleviated liver injury, reduced hepatocyte apoptosis, and promoted hepatocyte regeneration in ALF mice (Figure 6b–e).

### 2.9 hUCMSC‐sEV miRNA sequencing and bioinformatic analysis

To further explore the specific mechanisms of hUCMSC‐BAL, we performed high‐throughput sequencing of hUCMSC‐sEV (Figure S5a). The results showed that hUCMSC‐sEV were rich in a variety of miRNAs, among which miR‐139‐5p was the most abundant (Figure S5b). Functional enrichment can better determine the dominant pathway controlled by hUCMSC‐sEV. GO analysis of the targets genes of the top 10 miRNAs showed that “regulation of transcription from RNA polymerase II promoter” and “protein phosphorylation” in the biological process component (Figure S5c), “nucleoplasm” and “cytosol” in the cellular component (Figure S5d), and “protein binding” and “transcription factor activity, sequence‐specific DNA binding” in the molecular function component had the highest significance (Figure S5e). KEGG pathway enrichment was dominated by the Rap1, MAPK, and cAMP signaling pathways (Figure S5f). In summary, these results indicate a possible contribution of hUCMSC‐sEV miRNAs to the inhibition of hepatocyte apoptosis.

### 2.10 Conservatism of miR‐139‐5p expression

**FIGURE 7:**
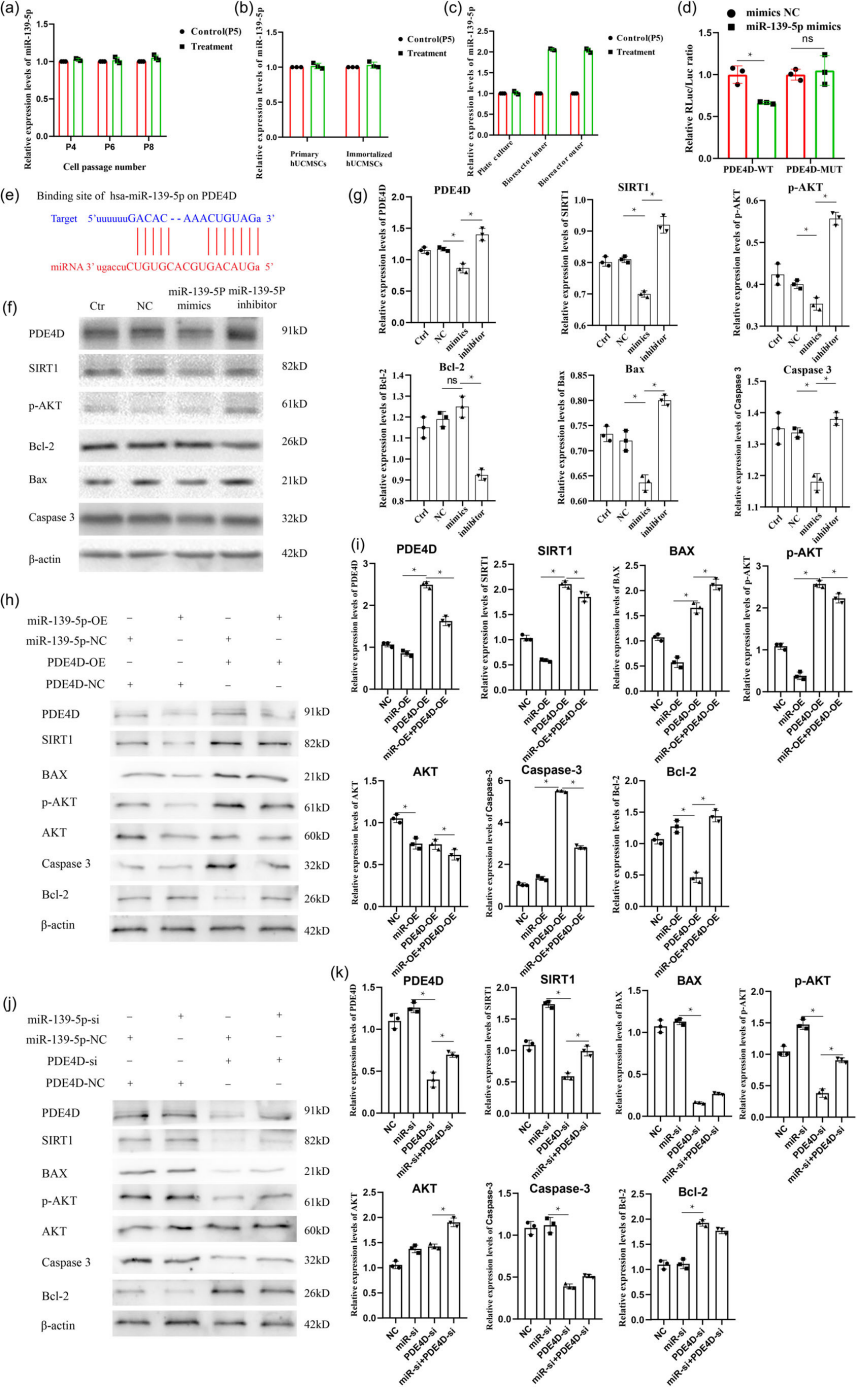
sEV miR‐139‐5p/PDE4D axis plays an important role in ALF treatment. (a) miR‐139‐5p expression of different cell passage numbers (compared with passage 5). (b) miR‐139‐5p expression of different sources (compared with passage 5). (c) miR‐139‐5p expression of different culture methods (compared with passage 5). (d) The binding relationship between PDE4D and miR‐139‐5p was verified using dual‐luciferase assay. (e) The binding sites between miR‐139‐5p and PDE4D. (f) Western blotting of PDE4D, SIRT1, p‐AKT, Bcl‐2, Bax, and Caspase 3 in L02 cell ALF model transfected with miR‐139‐5p mimics, miR‐139‐5p inhibitor, or NC. (g) Relative protein levels of PDE4D, SIRT1, p‐AKT, Bcl‐2, Bax, and Caspase 3 in L02 cell ALF model transfected with miR‐139‐5p mimics, miR‐139‐5p inhibitor, or NC; (h) Western blotting of PDE4D, SIRT1, AKT, p‐AKT, Bcl‐2, Bax, and Caspase 3 in L02 cell ALF model transfected with miR‐139‐5p‐OE, PDE4D‐OE, miR‐139‐5p‐OE + PDE4D‐OE, or NC; (i) Relative protein levels of PDE4D, SIRT1, AKT, p‐AKT, Bcl‐2, Bax, and Caspase 3 in L02 cell ALF model transfected with miR‐139‐5p‐OE, PDE4D‐OE, miR‐139‐5p‐OE + PDE4D‐OE, or NC; (j) Western blotting of PDE4D, SIRT1, AKT, p‐AKT, Bcl‐2, Bax, and Caspase 3 in L02 cell ALF model transfected with miR‐139‐5p‐si, PDE4D‐si, miR‐139‐5p‐si + PDE4D‐si, or NC; (k) Relative protein levels of PDE4D, SIRT1, AKT, p‐AKT, Bcl‐2, Bax, and Caspase 3 in L02 cell ALF model transfected with miR‐139‐5p‐si, PDE4D‐si, miR‐139‐5p‐si + PDE4D‐si, or NC. OE: Overexpression; si: silence.

To clarify the conserved expression of miR‐139‐5p and provide a basis for subsequent clinical application, we detected the expression of miR‐139‐5p in hUCMSCs from different passages, culture methods, and sources. The results showed that miR‐139‐5p was stably expressed by hUCMSCs from different passages, culture methods, and sources (Figure 7a–c). Interestingly, miR‐139‐5p expression in the bioreactor was significantly higher than that in the plate culture (Figure 7c).

### 2.11 PDE4D was a direct target of miR‐139‐5p

The results of the target genes of miR‐139‐5p predicted by online miRNA target gene prediction websites showed that PDE4D, LCOR, ZBTB34, NR5A2, and DCBLD2 were the main target genes (Figure S6). Among them, PDE4D can regulate the phosphorylation of Akt, which is known to inhibit several pro‐apoptotic Bcl‐2 family members, such as Bax.^29^ Moreover, by binding site prediction, we found that miR‐139‐5p bound to the PDE4D 3′‐UTR. Furthermore, the results of dual‐luciferase reporter assays also confirmed this observation (Figure 7d,e).

### 2.12 sEV miR‐139‐5p/PDE4D axis plays an important role in ALF treatment

To further explore the effect of the miR‐139‐5p/PDE4D axis on ALF treatment, we used L02 cells and HepaRG cells to construct ALF models and then transfected them with miR‐139‐5p mimics, miR‐139‐5p inhibitors, and miR‐negative control (NC). The results showed that miR‐139‐5p could significantly reduce the expression levels of PDE4D, SIRT1, p‐AKT, Bax and Caspase 3 as well as significantly increase the expression level of Bcl‐2 (Figures 7f,g and S7a,b).

**FIGURE 8:**
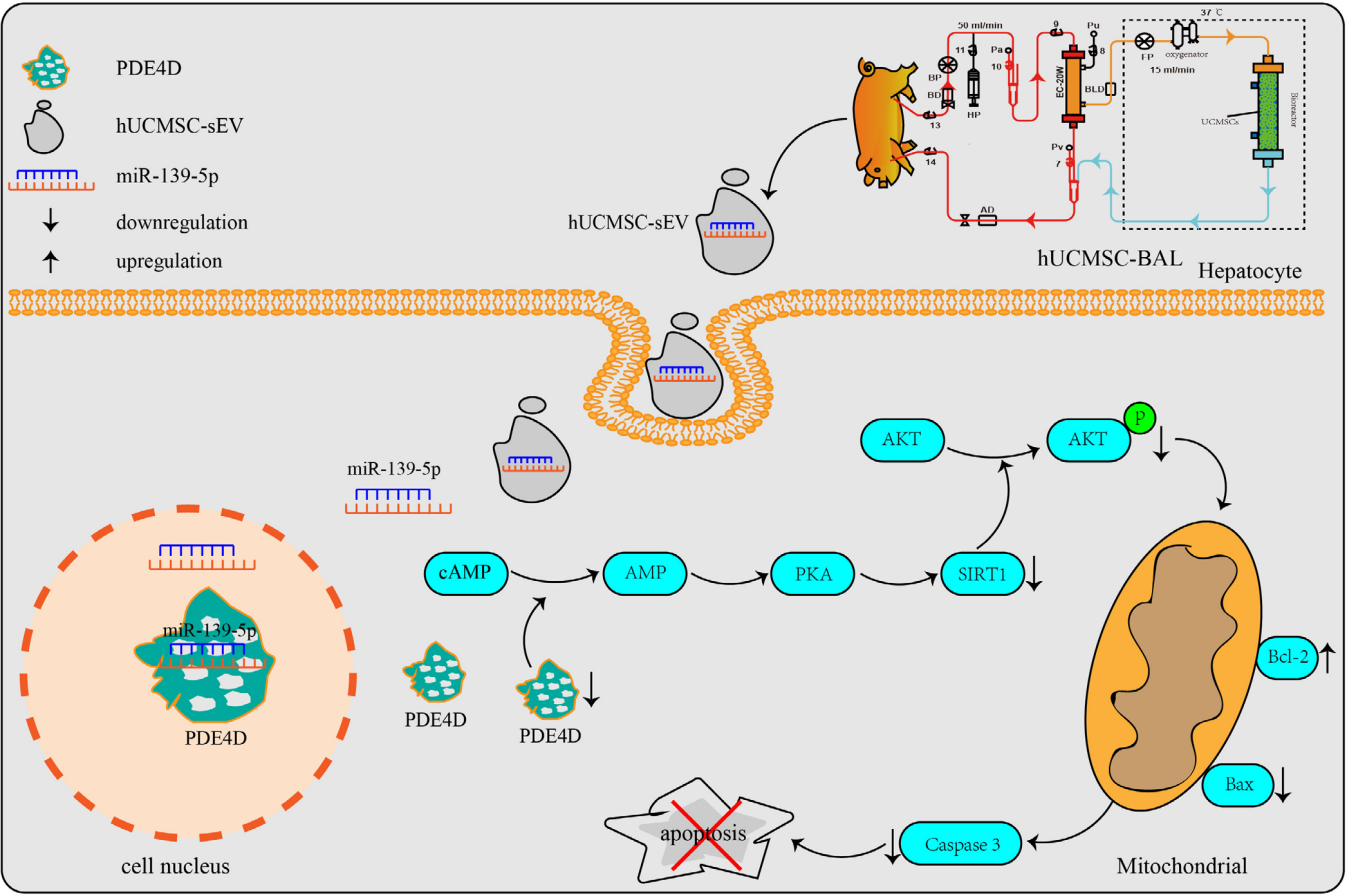
Schematic diagram of the mechanism of hUCMSC‐BAL in ALF treatment. hUCMSC‐BAL releases miR‐139‐5p‐containing sEV, which are endocytosed and taken up by hepatocytes to activate the miR‐139‐5p/PDE4D/SIRT1/Bcl‐2 pathway to inhibit hepatocyte apoptosis.

Furthermore, we constructed UCMSCs with stable overexpression or knockdown of miR‐139‐5p and L02 and HepaRG cell lines with stable overexpression or knockdown of PDE4D. The results shown that the sEV miR‐139‐5p/PDE4D axis plays an important role in ALF treatment (Figures 7h–k and S7c,d). Therefore, we believe that hUCMSC‐BAL may improve ALF liver injury by inhibiting hepatocyte apoptosis through the sEV miR‐139‐5p/PDE4D/SIRT1/Bcl‐2 pathway (Figure 8).

## 3 DISCUSSION

In this study, we successfully constructed an hUCMSCs‐based bioreactor and verified it using a porcine ALF model. The results showed that the hUCMSCs bioreactor significantly improved liver function in ALF, reduced inflammation and liver cell death, and prolonged ALF animal survival time. The pathogenesis of ALF is different at each stage; therefore, it is necessary to select appropriate treatment methods according to the relevant pathological changes. Our study demonstrated that the hUCMSCs bioreactor can significantly reduce liver cell death in the early stage of ALF by regulating the homeostasis and immune environment around normal liver cells through sEV, and that the sEV miR‐139‐5p/PDE4D axis may play an important role in this. In the middle and late stages of ALF, hepatocyte functional replacement therapy and homeostasis regulation can be used simultaneously for treatment.

Selection of the optimal time to begin treatment and duration of treatment is an important step in bioartificial liver therapy that has been debated in previous studies. For example, previous studies have reported optimal times to begin treatment of 2 h,^30^ 12 h,^13^ 24 h,^14^, ^17^ 36 h,^31^ and 48 h^12^, ^15^ after modeling, and optimal treatment durations of 3 h,^14^, ^17^ 6 h,^12^, ^13^, ^31^ 8 h,^15^, ^32^ and 12 h.^33^ Theoretically, the earlier the treatment begins, the longer the treatment duration and the better the treatment effect. In this study, D‐gal was used to induce the ALF model; therefore, the timing of bioartificial liver treatment should be selected after the drug is completely metabolized. A previous study showed that D‐gal was completely absorbed 12 h after injection^14^; thus, in our study, hUCMSC‐BAL therapy was started 12 h after D‐gal injection and continued for 8 h.

The seed cells and bioreactors are the two most important elements in bioartificial livers. At present, many seed cells are related to liver cell functional replacement therapy, including human hepatocytes,^34^ liver tumor cell lines,^10^ human‐induced hepatocytes,^14^, ^16^ and porcine hepatocytes.^11^, ^15^ However, their application is limited by the limited sources of human liver cells, tumorigenicity, and safety and ethical concerns. Conversely, hUCMSCs have the advantages of wide availability, low immunogenicity, few ethical restrictions, and high safety, and are expected to become important new seed cells. Bioreactors, as the other key element, provide a good environment for the growth and metabolism, substance exchange, and immune isolation of seed cells. Previous studies have shown that hollow fiber bioreactors have the disadvantage of insufficient oxygenation, which is not conducive to the growth of commonly used seed cells.^35^ However, because the tissue oxygen concentration of hUCMSCs is less than 5%, hypoxic culture in vitro can effectively simulate the in vivo microenvironment and help maintain the proliferation, metabolism, and other physiological processes of hUCMSCs. Therefore, the low‐oxygen environment of a hollow‐fiber bioreactor is beneficial for the growth and proliferation of hUCMSCs. In this study, we constructed an hUCMSC bioreactor that can amplify hUCMSCs on a large scale (by up to 10^11^) for clinical treatment, while maintaining the activity and function of hUCMSCs. Moreover, we detected the content of sEV proteins in the culture medium of the hollow fibers inside and outside the cavity, and the results showed no significant difference. hUCMSC‐BAL was constructed to avoid adverse reactions caused by hUCMSCs infusion in patients and to allow hUCMSCs derivatives (sEV and free factors) to enter patients through the bioreactor semi‐permeable membrane to play a therapeutic role. Therefore, hUCMSC‐BAL therapy compensates for the difficulties and deficiencies in hUCMSCs transplantation and the isolation and purification of hUCMSCs‐derived sEV.

HE is a syndrome characterized by impaired brain function caused by acute and chronic liver disease.^36^ Although the pathophysiology of HE is not fully understood, it is linked to the presence of inflammatory mediators and Amm.^37^ Impaired detoxification due to ALF leads to the accumulation of multiple toxins, which then induce the release of inflammatory cytokines, eventually leading to multiorgan failure.^38^ Sepsis with multiorgan failure and HE are the two major causes of death in patients with ALF.^38^ In our study, HE syndrome was observed in group A at 48 h and in group B at 60 h, but not in group C. Furthermore, the levels of Amm, TBIL, and inflammatory mediators in group C after hUCMSC‐BAL treatment were significantly lower than those in groups A and B. These results showed that hUCMSC‐BAL can significantly reduce the levels of inflammatory mediators, improve the microenvironment of normal hepatocytes, avoid cytokine storms, protect the detoxification of hepatocytes, and prevent multiorgan failure and HE.

sEV are a type of EV formed through an endocytic process and released through a multivesicular body into the extracellular space; they play important roles in cell–cell communication involved in the regulation of a range of biological processes.^39^, ^40^, ^41^ It is becoming increasingly clear that sEV derived from MSCs have a protective effect on ALF liver injury.^42^, ^43^, ^44^ In our study, we found that hUCMSC‐BAL could release sEVs into the body through a bioreactor semi‐permeable membrane to rescue ALF. To explore the underlying mechanisms, we extracted sEV from the hUCMSCs‐based bioreactor to treat the mouse ALF model and found that these sEV had an obvious protective effect on liver injury. Furthermore, high‐throughput sequencing of hUCMSC‐sEV showed that they are rich in a variety of miRNAs, among which miR‐139‐5p was the most abundant. Finally, we predicted the target genes of miR‐139‐5p and found that PDE4D was one of the main target genes that could inhibit cell apoptosis.

To further explore the effect of the miR‐139‐5p/PDE4D axis in ALF treatment, we used L02 and HepaRG cells to construct ALF models and then transfected them with miR‐139‐5p mimics, miR‐139‐5p inhibitors, and miR‐NC. The results showed that miR‐139‐5p could significantly reduce the expression levels of PDE4D, SIRT1, p‐AKT, Bax, and Caspase 3 as well as significantly increase the expression level of Bcl‐2. Therefore, we believe that hUCMSC‐BAL may improve ALF liver injury by inhibiting hepatocyte apoptosis through the sEV miR‐139‐5p/PDE4D/SIRT1/Bcl‐2 pathway. Furthermore, we constructed UCMSCs with stable overexpression or knockdown of miR‐139‐5p and L02 and HepaRG cell lines with stable overexpression or knockdown of PDE4D. The results showed that the sEV miR‐139‐5p/PDE4D axis plays an important role in ALF treatment.

However, our study has some limitations. First, the small number of experimental animals may lead to certain errors owing to the small sample size; thus, the experimental results require further verification by increasing the sample size. Second, clinical ALF is an extremely complex disease, and the ALF model induced by D‐gal may not completely simulate clinical ALF; thus, further clinical verification is required. Third, the time required for the large‐scale expansion of hUCMSCs to the number of clinical treatments is relatively long; thus, further study should attempt to shorten the amplification time. Fourth, subject to experimental conditions we only use male pigs in our manuscript which may produce some deviation due to gender. Lastly, although we report the therapeutic effects of hUCMSC‐BAL treatment, probably via sEV release, as well as the important role of the sEV miR‐139‐5p/PDE4D axis, the related mechanisms require further research.

In conclusion, we successfully developed an in vitro bioartificial liver based on hUCMSCs, which can inhibit inflammatory responses, regulate homeostasis to reduce liver cell death, promote liver cell regeneration, and prolong the survival time of animals with ALF. We think that the sEV miR‐139‐5p/PDE4D axis plays an important role in this process. Further randomized clinical trials are required to verify whether hUCMSC‐BAL treatment has therapeutic benefits in patients with ALF.

## 4 MATERIALS AND METHODS

### 4.1 Animals

Fifteen male miniature pigs, 0.2–1.0 years old and weighing 20–30 kg, were purchased from Dongguan Songshan Lake Pearl Laboratory Animal Science and Technology Co., Ltd., license No. SCXK (Guangdong) 2017–0030. Twelve male C57/BL6 mice, 10–15 weeks old and weighing 25–35 g, were purchased from the Experimental Animal Center of Southern Medical University. Mice were kept in cages at 22 ± 1°C and exposed to light for 12 h (from 06:00 to 18:00) and dark for 12 h. All experimental animals were isolated and caged, fed with special food, and allowed to drink freely. All animals were fed adaptively for 1 week and then fasted for 12 h before experimentation. Animal experiments were approved by the Animal Ethics Committee of Zhujiang Hospital, Southern Medical University (LACE‐2020‐155).

### 4.2 hUCMSCs isolation, culture, identification, and amplification

#### 4.2.1 Isolation and culture of hUCMSCs

After obtaining approval from the Ethics Committee of Zhujiang Hospital and patient consent, fresh umbilical cord was obtained from puerpera. The isolation and culture of hUCMSCs were performed according to a previously described method.^45^ Briefly, umbilical cord tissue was subjected to type I collagenase digestion, centrifugation, filtration, and other steps until hUCMSCs were obtained. The culture medium was changed every 3 days. The cells were passaged upon reaching 80% confluency(Figure 1b). To ensure cell viability, this experiment only used cells from passages 3–5.

#### 4.2.2 Flow cytometry

A suspension of 1 × 10^6^ hUCMSCs (100 μL) was placed in a centrifuge tube and the following antibodies were used for fluorescence labeling: CD34, CD45, CD73, CD90, CD19, CD11b, CD105, and HLA‐DR (Table S1). After incubation for 30 min in the dark, the cells were washed twice with phosphate‐buffered saline (PBS), and flow cytometry (BD Bioscience, BD FACSCalibur) was performed to detect the abovementioned marker proteins.

#### 4.2.3 Differentiation ability of hUCMSCs

According to the manufacturer's instructions, adipogenic induction medium (STEMCELL, Catalog #05412), osteogenic induction medium (STEMCELL, Catalog #05465), and chondrogenic induction medium (STEMCELL, Catalog #05455) were used to culture hUCMSCs. After 21 days, Oil red O, alizarin red, and safranin O staining was performed to evaluate the ability of hUCMSCs to differentiate into adipocytes, osteocytes, and chondrocytes, respectively.

#### 4.2.4 Large‐scale amplification of hUCMSCs in the hollow fiber bioreactor

The hUCMSC bioreactor comprises a hollow fiber bioreactor, peristaltic pump, liquid storage bottle, and connecting pipeline (Figure 1a). The identified hUCMSCs were first inoculated into plate culture bottles, expanded to 10^8^ cells using customized serum‐free medium, and then inoculated into the bioreactor culture system. As the rate of blood GLU decline is directly proportional to the number of cells in the bioreactor, the culture medium should be changed regularly according to the sugar level of the circulating culture medium (when the sugar level is less than 1.5 mmoL/L or the culture time is > 5 days) until the hUCMSCs are amplified to 10^9^–10^10^. The culture medium in the inner and outer chambers of the bioreactor was collected and stored in the refrigerator at −80°C for standby application.

### 4.3 hUCMSC‐BAL rescues porcine ALF model

#### 4.3.1 Study design

Fifteen miniature pigs (Table S2) were randomly divided into three groups (A, B, C) after central venous catheterization. Then, D‐gal at 0.40 g/kg body weight was injected through a central venous catheter to generate the ALF model. The control group received standard therapy (ST) and the experimental group received low‐dose hUCMSC‐BAL + ST and high‐dose hUCMSC‐BAL + ST for 8 h at 12 h after modeling. The grouping was as follows (Figure 2a):

Group A (*n* = 5): 0.40 g/kg D‐gal+ ST;
Group B (*n* = 5): 0.40 g/kg D‐gal+ low dose hUCMSC‐BAL (10^9^) + ST;
Group C (*n* = 5): 0.40 g/kg D‐gal+ high‐dose hUCMSC‐BAL (10^10^) + ST.

#### 4.3.2 Establishment of the drug‐induced porcine ALF model

After 1 week of adaptive feeding, the experimental animals were anesthetized (pentobarbital sodium (30 mg/kg) and sumianxin II (0.1 mL/kg)). The modified Seldinger puncture method was used to insert indwelling double‐lumen hemodialysis catheters (11.5 F, 20 cm, ABLE) into the internal jugular vein and femoral vein, respectively. These catheters were then used for blood collection, maintenance of anesthesia, fluid infusion, and BAL treatment pathway construction. A large dose of D‐gal did not provide a sufficient treatment window, and a small dose did not induce ALF, but allowed the animal to recover by itself. Therefore, according to previous studies^14^, ^46^ and our previous research results,^15^ we selected a dose of 0.40 g/kg to construct the ALF model. After injection of D‐gal (*t* = 0 h), the catheter was closed with heparin saline (2500 U/ml) to keep it unobstructed and allow the animals to wake up naturally from anesthesia. Clinical observations were conducted every 6 h during the experiment. Blood samples were collected every 12 h for blood detection (albumin [ALB], AST, ALT, blood urea nitrogen [BUN], TBIL, CRE, GLU, Amm, etc.) and coagulation function assessment (Drim‐7000i, FUJI, and URIT). All samples were stored at −80°C for further testing. Animals in all groups received 500 mL of normal saline after D‐gal injection. When the animals stopped feeding and drinking because of ALF, they received 5% dextrose normal saline (DNS5) at 50 mL/h to satisfy their basic water and energy needs. Twenty milliliters of 50% dextrose was injected when the blood GLU level fell below 5 mmol/L. The experimental timeline is shown in Figure 2a. The study endpoint was animal death or survival for 168 h.

#### 4.3.3 hUCMSC‐BAL treatment

hUCMSC‐BAL treatment was initiated 12 h after D‐gal administration for 8 h. Pentobarbital sodium and sumianxin II were used to induce basal anesthesia. Propofol was injected via a central venous catheter (0.15–0.20 mg/kg/min) to maintain slight anesthesia, and the blood pressure, heart rate, and respiratory rate were monitored during hUCMSC‐BAL treatments. The hUCMSC‐BAL device was connected to the ALF model pigs via a dual‐chamber hemodialysis catheter inserted into the jugular and femoral veins (Figures 2b and S1). Saline (500 mL) was injected before the treatment to prevent hypotension. Blood chemistry and coagulation function tests were performed every 2 h during treatment. Heparin was injected to maintain the APTT between 175 and 250 s.

#### 4.3.4 Inflammatory factor detection

All blood samples were collected, centrifuged at 4000 rpm for 10 min to collect plasma, and stored at −80°C in a refrigerator. Cytokines were detected by ELISA kit (Table S3).

#### 4.3.5 Histopathology and immunohistochemistry

After the experimental animals died or reached the study endpoint, animal tissue samples (heart, liver, spleen, lung, kidney, large intestine, small intestine, and brain) were collected, fixed, embedded in paraffin, stained with H&E, and the liver tissue was stained by immunohistochemistry. Ki‐67 and TUNEL staining was conducted according to the manufacturer's instructions. Professional pathologists used the Suzuki classification system to observe and score H&E sections of the liver tissue. The score range was 0–4 points for liver parenchymal necrosis, hepatic sinus congestion, and cytoplasmic vacuolization. Each pathologist examined three liver sections from each animal and randomly selected three visual fields from each section for scoring. The average score of each animal was determined by the sum of all scores. The positive results of Ki‐67 and TUNEL staining were counted and defined as the regeneration and apoptosis indices, respectively. Masson, Sirius red, PAS, and Oil red staining was also performed.

#### 4.3.6 Transmission electron microscopy

The obtained liver tissue was divided into 1 × 1 cm tissue blocks and fixed in glutaraldehyde + paraformaldehyde electron microscopy solution for 10 min. The tissue blocks were then divided into 2 × 2 mm blocks and fixed in electron microscopy solution. After overnight dehydration at 4°C, the slices were scanned using a transmission electron microscope.

### 4.4 hUCMSC‐sEV extraction and identification

Differential ultracentrifugation was used to extract the sEV. Briefly, centrifugal forces of 300, 2000, 10,000, and 100,000×g were used to centrifuge the cell culture medium. The final pellet was resuspended in 200 μL of PBS. Transmission electron microscopy was used to observe the shape of the sEV. NanoView was used to measure the concentration and particle‐size distribution of sEV. Western blotting was used to measure the positive protein markers CD9, TSG101, CD81, syntenin, and CD63, and the sEV negative protein marker calnexin after employing a BCA protein quantification kit to determine the protein concentration of sEV according to the manufacturer's instructions (Table S1).

### 4.5 Fluorescence imaging tracing of sEV in vitro and in vivo

To identify the transport of sEV in vitro and in vivo, sEV were labeled with DIR or PHK26 fluorescent dye. DIR‐labeled sEV were injected through the tail vein of mice, and their transport in vivo was observed within 48 h. PBS was injected as a control. PHK26‐labeled sEV were added to the medium of L02 and AML‐12 cells and observed under a fluorescence microscope within 48 h.

### 4.6 hUCMSC‐sEV treatment of the mouse ALF model

Mice in the experimental group (sEV group) and control group (PBS group) were intraperitoneally injected with D‐gal (800 mg/kg, Sigma‐Aldrich) and LPS (50 μg/kg, Sigma‐Aldrich), respectively, to construct the ALF model. The sEV group was injected with hUCMSC‐sEV via the tail vein 24 h before modeling, whereas the PBS group was injected with the same amount of PBS solution. The mice were sacrificed 1 and 3 h after modeling, and samples were collected for the relevant tests.

After anesthesia, cardiac blood was collected from the mice, allowed to stand for 2 h, and then centrifuged at 3500 rpm for 10 min. The supernatant was retained and stored at −80°C for later use. Serum ALP, ALT, AST, and BUN levels were determined using a dry biochemical analyzer (Catalyst One; IDEXX Laboratory).

After the experimental animals died or reached the study endpoint, animal liver tissue samples were collected, fixed, embedded in paraffin, and stained with H&E, and the liver tissue was stained with the relevant immunohistochemistry stains (Ki‐67 and TUNEL) according to the manufacturer's instructions.

### 4.7 hUCMSC‐sEV transcriptome assay and data analysis

High‐throughput sequencing and OE Biotech were used to determine the miRNA expression profiles. Total RNA was extracted and quantified, and a cDNA library was constructed after removing ribosomal RNA. An Illumina sequencer (HiSeqTM 2500) was used after quality inspection. Finally, miRNAs were confirmed and statistically analyzed.

The clusterProfiler package was used to perform functional annotation and enrichment analysis of KEGG and GO data for the top 10 most abundant miRNA target genes. For the three major categories in the GO database (biological processes, cell components, and molecular functions) and KEGG pathways, the top 10 most significant terms/pathways were displayed.

### 4.8 Detection of the conservation of miR‐139‐5p expression

An miRNeasy Kit (50 reactions) (Qiagen) was used to extract total RNA, which was then reverse‐transcribed using an miRNA 1st strand cDNA synthesis Kit (AG). The target sequence was amplified by qRT‐PCR using a SYBR Green PCR kit (AG11702). The 2−ΔΔCt method was used to determine the relative fold changes in target gene expression in the EVs normalized to the levels in the corresponding control EVs (defined as 1.0). U6 small nuclear RNA was used as an internal control for the miRNA and mRNA assays. All experiments were performed in duplicate. PCR primers for miR‐139‐5p and U6 (Table S4) were purchased from RiBoBio.

### 4.9 Predicting the target gene of miR‐139‐5p

The online miRNA target gene prediction resource miRWalk^47^ was used to predict the target genes of miR‐139‐5p, with a 95% confidence interval and 3′‐UTR as the parameters, respectively. The intersection of the three datasets was then used to obtain the final main target genes.

### 4.10 Dual‐luciferase reporter assay

Dual‐luciferase reporter gene assay was used to detect the binding sites between miR‐139‐5p and PDE4D. The corresponding PDE4D sequences of luciferase reporter plasmids, WT, MUT, and mimic‐NC or miR‐139‐5p mimic were constructed as described previously,^48^ and were co‐transfected into 293T cells, which were collected from each well after 48 h. The potency or activity of luciferase was detected through the synergy 2.

### 4.11 Construction of L02 cell ALF model and treatment with miR‐139‐5p mimics or inhibitor

L02 cells (1 × 10^5^) were inoculated into 6‐well plates; transfected with miR‐139‐5p mimics, miR‐139‐5p inhibitors, and miR‐NC, according to the manufacturer's instructions; treated with 20 mmoL/L D‐gal 72 h after transfection; and harvested 12 h after D‐gal addition. The specific grouping is shown in Figure 7e. The miR‐139‐5p mimics, miR‐139‐5p inhibitors, and miR‐NC were purchased from Tsingke Bio Co., Ltd.

### 4.12 Construction of UCMSCs with stable overexpression or knockdown of miR‐139‐5p

UCMSCs with miR‐139‐5p overexpression or knockdown were constructed using the methods reported in the literature.^49^ The main steps were: (1) construction of miR‐139‐5p overexpression or knockdown vector, (2) vector identification, (3) lentivirus transfection, (4) viral titer determination, (5) infection complex value determination, and (6) screening of stably transfected cell lines. UCMSCs with stable overexpression or knockdown of miR‐139‐5p were finally obtained. UCMSCs with stable overexpression or knockdown of miR‐139‐5p were amplified in the bioreactor, and sEV were isolated from the culture medium. Finally, the L02 cell ALF model was treated with sEV.

### 4.13 Construction of L02 and HepaRG cell lines with stable overexpression or knockdown of PDE4D

L02 and HepaRG cell lines with PDE4D overexpression or knockdown were constructed using the methods described above. The obtained cells were then used to construct the ALF model, which was subsequently treated with sEVs isolated from UCMSCs with stable overexpression or knockdown of miR‐139‐5p.

### 4.14 Protein extraction and western blotting

The protein was extracted from L02 and HepaRG cells in each group. The relative protein expression in L02 and HepaRG cells was detected by western blotting as described previously.^15^, ^29^ The antibodies used are shown in Table S1.

### 4.15 Statistical analysis

All data are presented as the mean ± SD. Unpaired two‐tailed Student's *t*‐test and one‐way analysis of variance (ANOVA) were used to test for statistical significance. The Mantel–Cox log‐rank test was used to test for survival time. *p* < 0.05 was considered to indicate a statistically significant difference. All statistical analyses were performed using SPSS (version 21.0; SPSS, Inc.) and GraphPad Prism 8.

## AUTHOR CONTRIBUTIONS

**Lei Feng:** Conceptualization (lead); data curation (equal); formal analysis (equal); funding acquisition (equal); methodology (equal); software (equal); visualization (equal); writing – original draft (lead). **Yi Wang:** Conceptualization (equal); data curation (equal); formal analysis (equal); methodology (equal); software (equal); visualization (equal); writing – original draft (equal). **Yu Fu:** Data curation (equal); formal analysis (equal); methodology (equal); software (equal); visualization (equal); writing – original draft (supporting). **Adilijiang Yimamu:** Data curation (equal); formal analysis (equal); methodology (equal); software (equal); visualization (equal); writing – original draft (supporting). **Zeyi Guo:** Data curation (equal); methodology (equal); software (equal); visualization (equal); writing – original draft (supporting). **Chenjie Zhou:** Data curation (equal); formal analysis (equal); methodology (equal); visualization (supporting); writing – review and editing (supporting). **Shao Li:** Investigation (supporting); methodology (supporting); project administration (equal); resources (equal). **Linya Zhang:** Project administration (equal); resources (supporting); software (equal); validation (supporting). **Jiasheng Qin:** Data curation (supporting); formal analysis (supporting); methodology (supporting); software (supporting); visualization (supporting). **Shusong Liu:** Data curation (supporting); formal analysis (supporting); project administration (supporting); resources (supporting); visualization (supporting). **Xiaoping Xu:** Methodology (supporting); supervision (supporting); writing – review and editing (supporting). **Zesheng Jiang:** Project administration (supporting); supervision (supporting); writing – review and editing (supporting). **Shaoru Cai:** Project administration (supporting); supervision (supporting). **Jianmin Zhang:** Methodology (supporting); resources (supporting); software (supporting). **Yang Li:** Project administration (supporting); resources (supporting). **Qing Peng:** Project administration (supporting); supervision (supporting). **Xiao Yi:** Conceptualization (supporting); methodology (supporting); supervision (supporting); validation (equal); writing – review and editing (equal). **Guolin He:** Conceptualization (equal); methodology (equal); resources (supporting); supervision (supporting); writing – review and editing (equal). **Ting Li:** Conceptualization (equal); data curation (supporting); formal analysis (supporting); funding acquisition (supporting); project administration (equal); writing – review and editing (equal). **Yi Gao:** Conceptualization (equal); funding acquisition (lead); project administration (lead); resources (equal); supervision (equal); writing – review and editing (equal).

## CONFLICT OF INTEREST STATEMENT

The authors declare no conflicts of interest.

### PEER REVIEW

The peer review history for this article is available at https://www.webofscience.com/api/gateway/wos/peer‐review/10.1002/btm2.10552.

## Supporting information


**Data S1:** Supporting Information

## Data Availability

The data used in this manuscript were available from the corresponding author on reasonable request.
