# Supplementary material for: A simple and efficient strategy for cell‐based and cell‐free‐based therapies in acute liver failure: hUCMSCs bioartificial liver
Source: Bioeng Transl Med. 2023 Jun 2;8(5):e10552. doi: 10.1002/btm2.10552 (PMC10486334; doi:10.1002/btm2.10552)
Supplement: Supplementary file 1 — Data S1: Supporting Information [file BTM2-8-e10552-s001.docx]

**SUPPORTING INFORMATION**


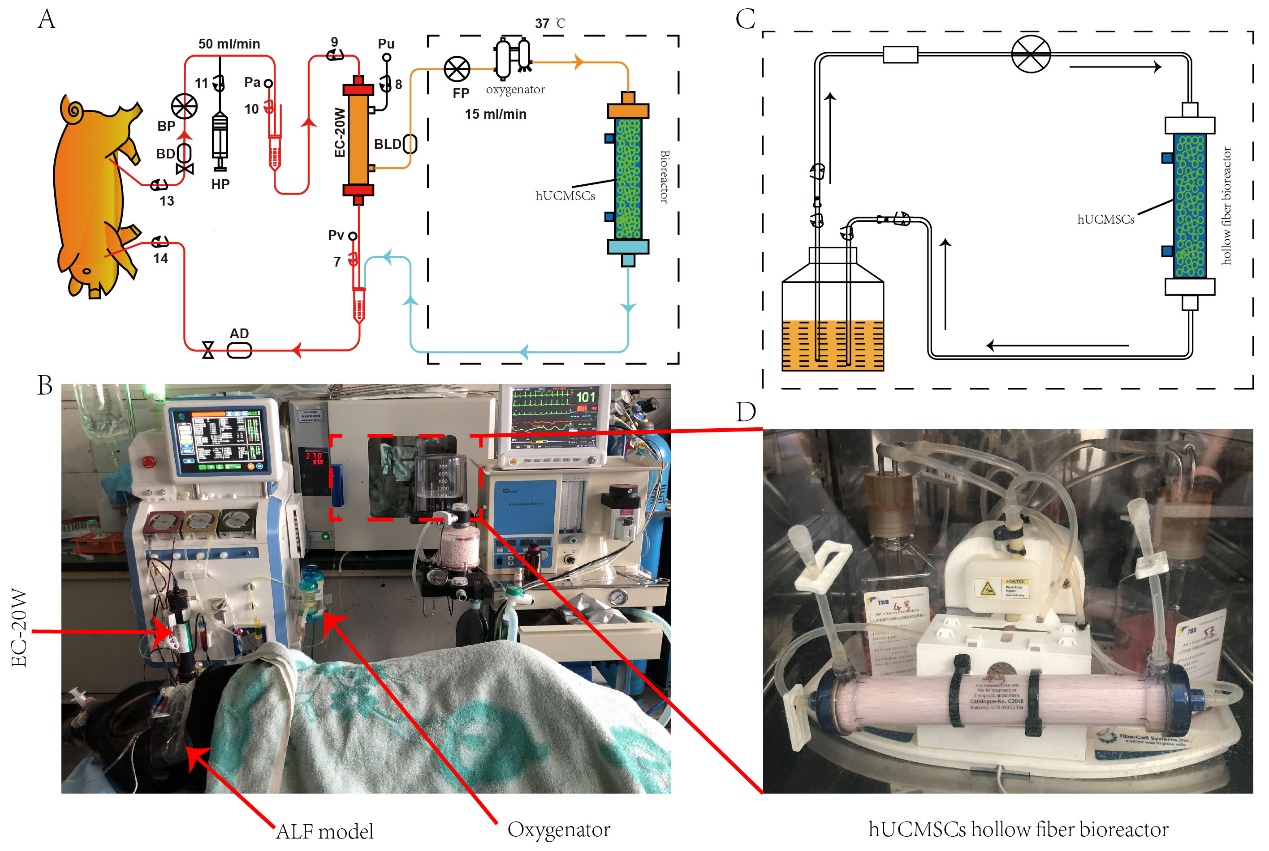


**Figure.S1. Schematic and physical drawings of hUCMSC-BAL and hollow fiber bioreactor.** A: Schematic drawings of hUCMSC-BAL; B: Physical drawings of hUCMSC-BAL; C: Schematic drawings of hollow fiber bioreactor; D: Physical drawings of hollow fiber bioreactor.


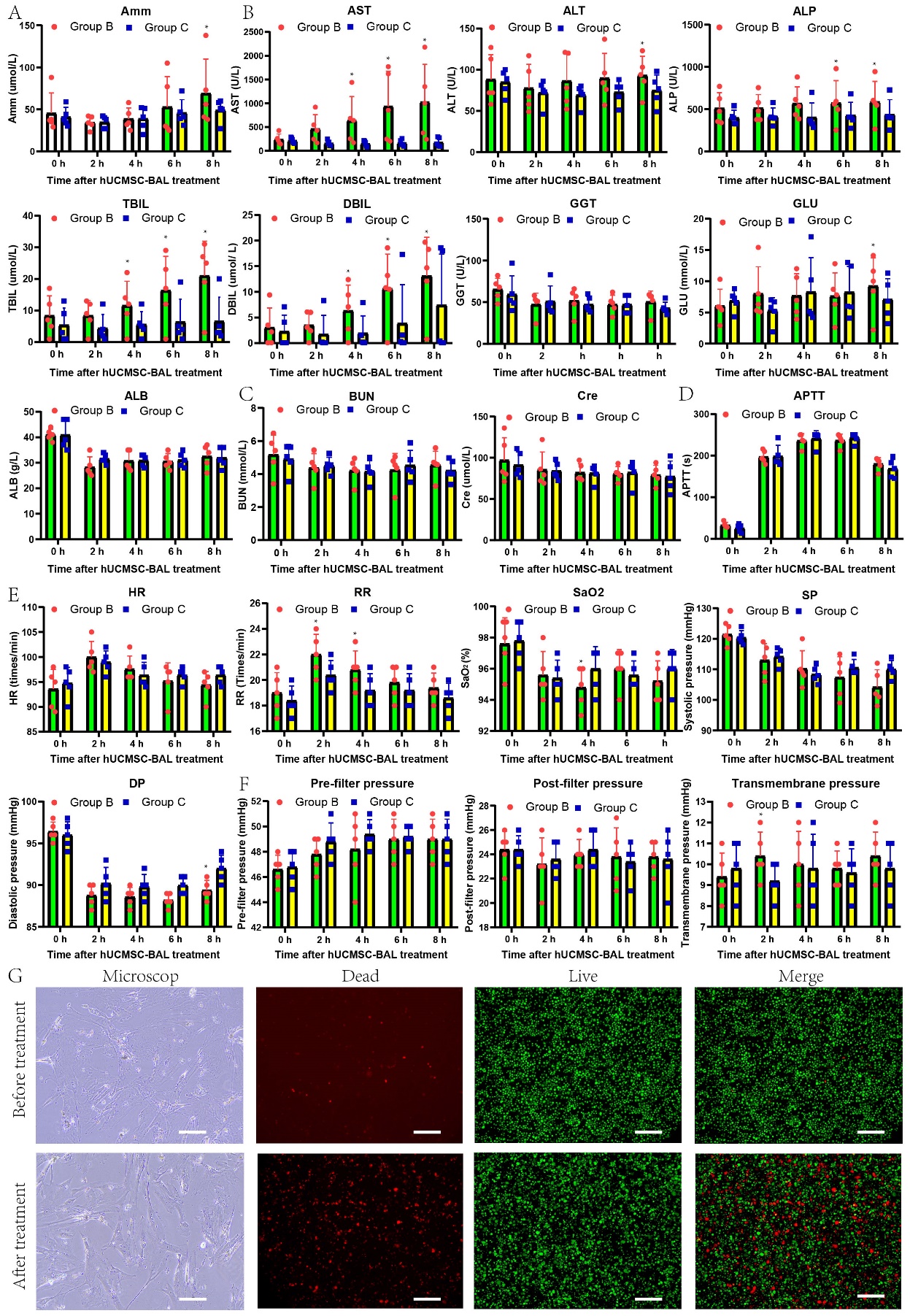


**Figure.S2.** Evaluation of hUCMSC-BAL during treatment. A: Dynamic changes of blood ammonia during treatment (n = 5 in each group). B: Dynamic changes of liver function indexes during treatment, i.e., plasma AST, ALT, DBIL, TBIL, ALP, ALB, GGT, and GLU (n = 5 in each group). C: Dynamic changes of renal function indexes during treatment, i.e., CRE and BUN (n = 5 in each group). D: Dynamic changes of APTT during treatment. E: Dynamic changes of vital signs during treatment, i.e., HR, RR, SaO_2_, SP, and DP (n = 5 in each group). F: Dynamic changes of pressure detected by hUCMSC-BAL during treatment (n = 5 in each group). G: Cell status of hUCMSCs in bioreactor before and after treatment. **P* < 0.05 by unpaired-tailed Student’s t-test. Group B: ALF+ST+low dose hUCMSC-BAL；Group C：ALF+ST+high dose hUCMSC-BAL. scale bar 200 μm.


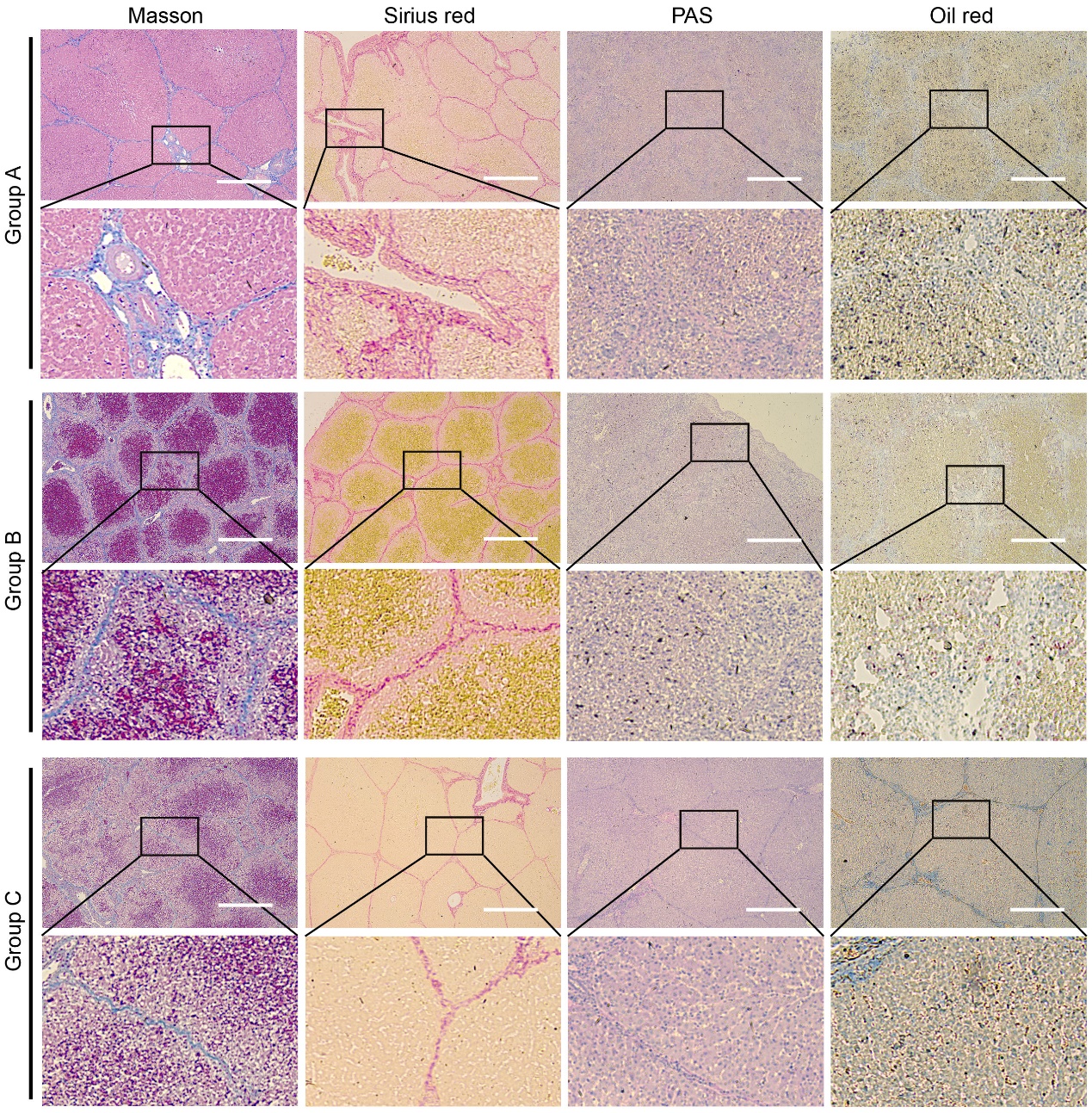


**Figure.S3. Special staining results of liver tissues in three groups** (higher magnification images are shown in the insets below)**.**  PAS: Periodic acid-schiff staining. scale bar 200 μm.


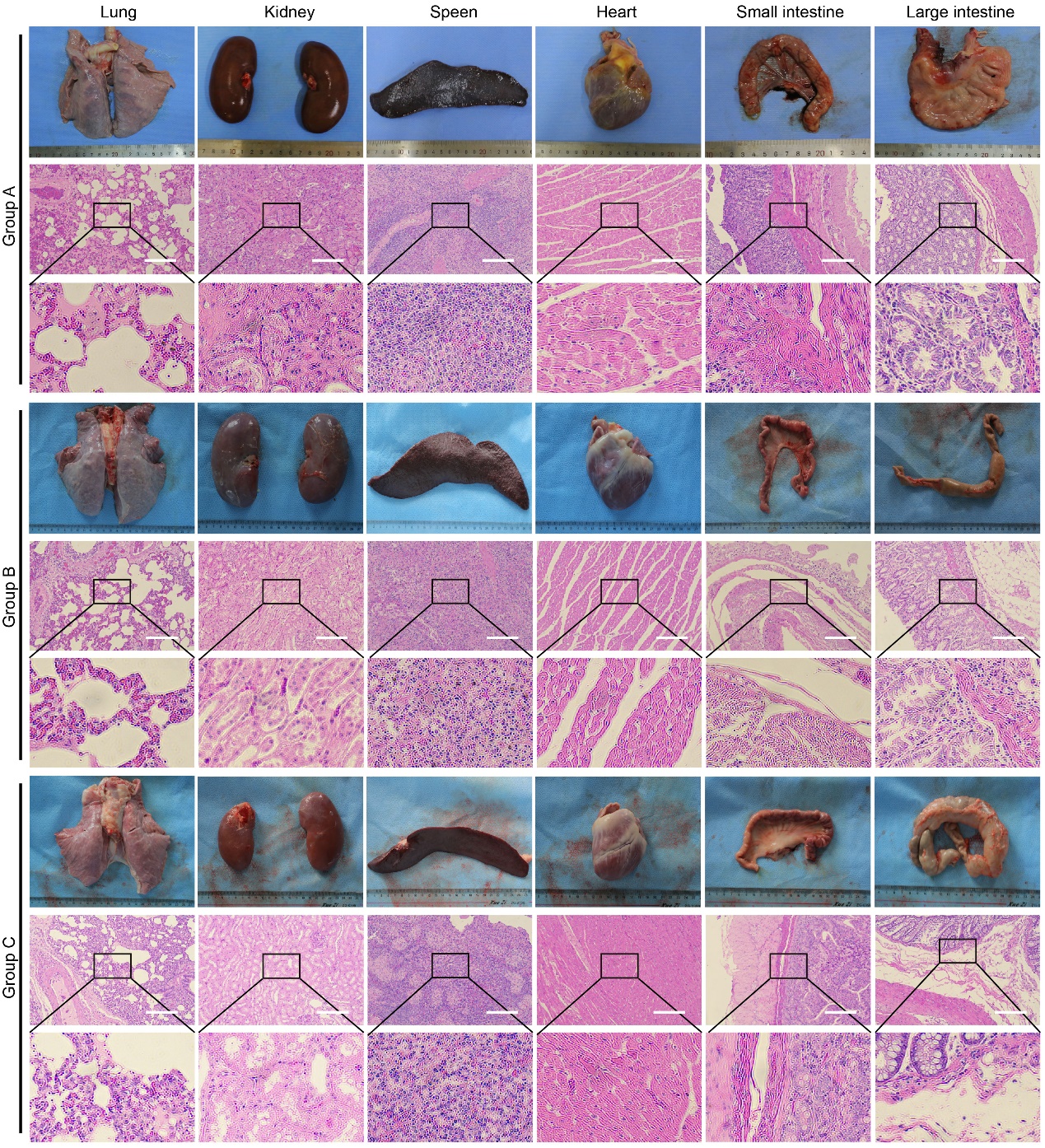


**Figure.S4.** Gross necroscopy specimens and H&E staining of extrahepatic organ in the three groups (higher magnification images are shown in the insets below). H&E: Hematoxylin-Eosin. scale bar 200 μm.


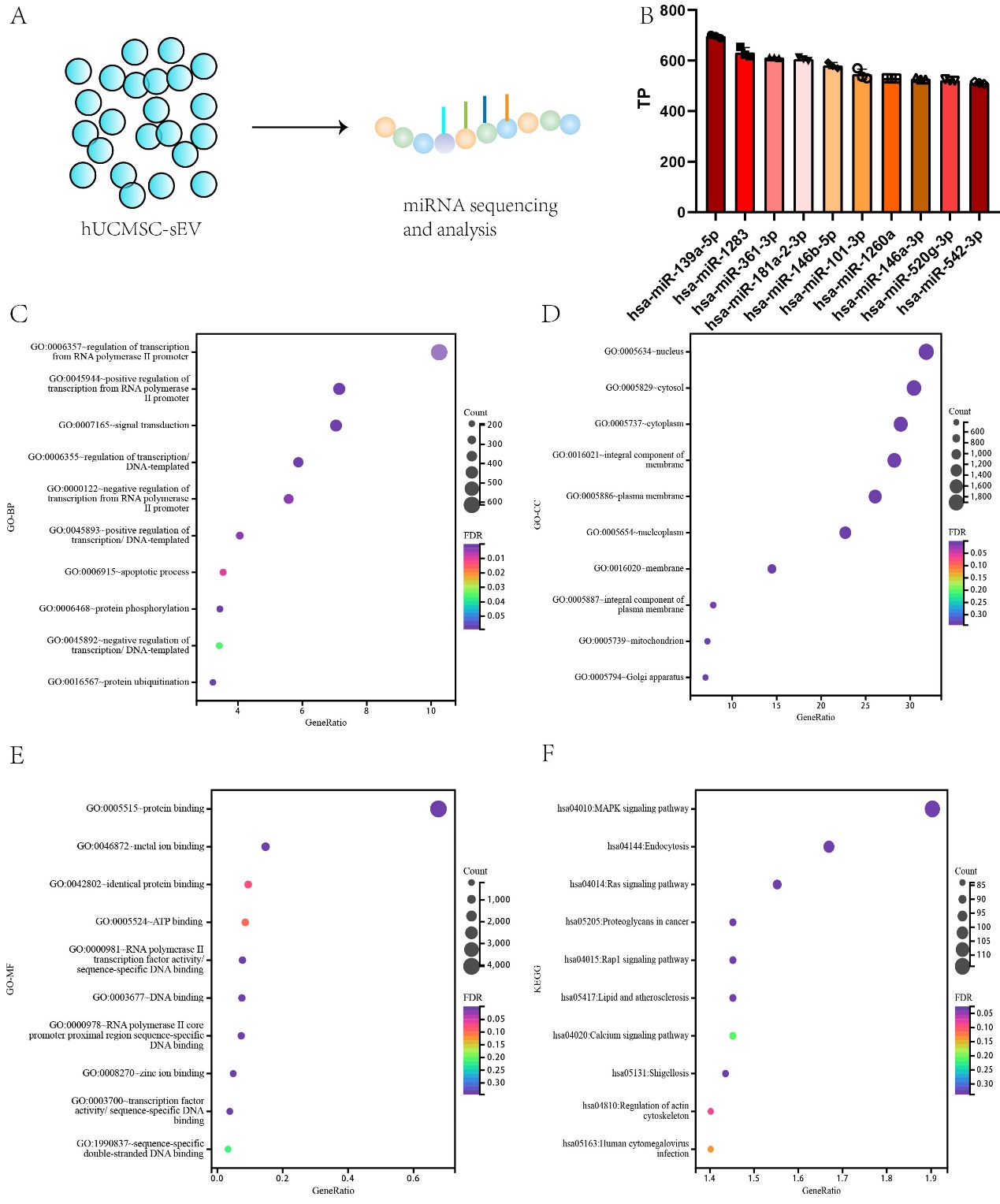


**Figure.S5.** **hUCMSC-sEV transcriptome detection and analysis.** A: Schematic diagram. B: Top 10 miRNA in hUCMSC-sEV. C: GO analysis (biological process, BP) in target genes of top 10 miRNA. D: GO analysis (cellular component, CC) in target genes of top 10 miRNA. E: GO analysis (molecular function, MF) in target genes of top 10 miRNA. F: KEGG analysis in target genes of top 10 miRNA.


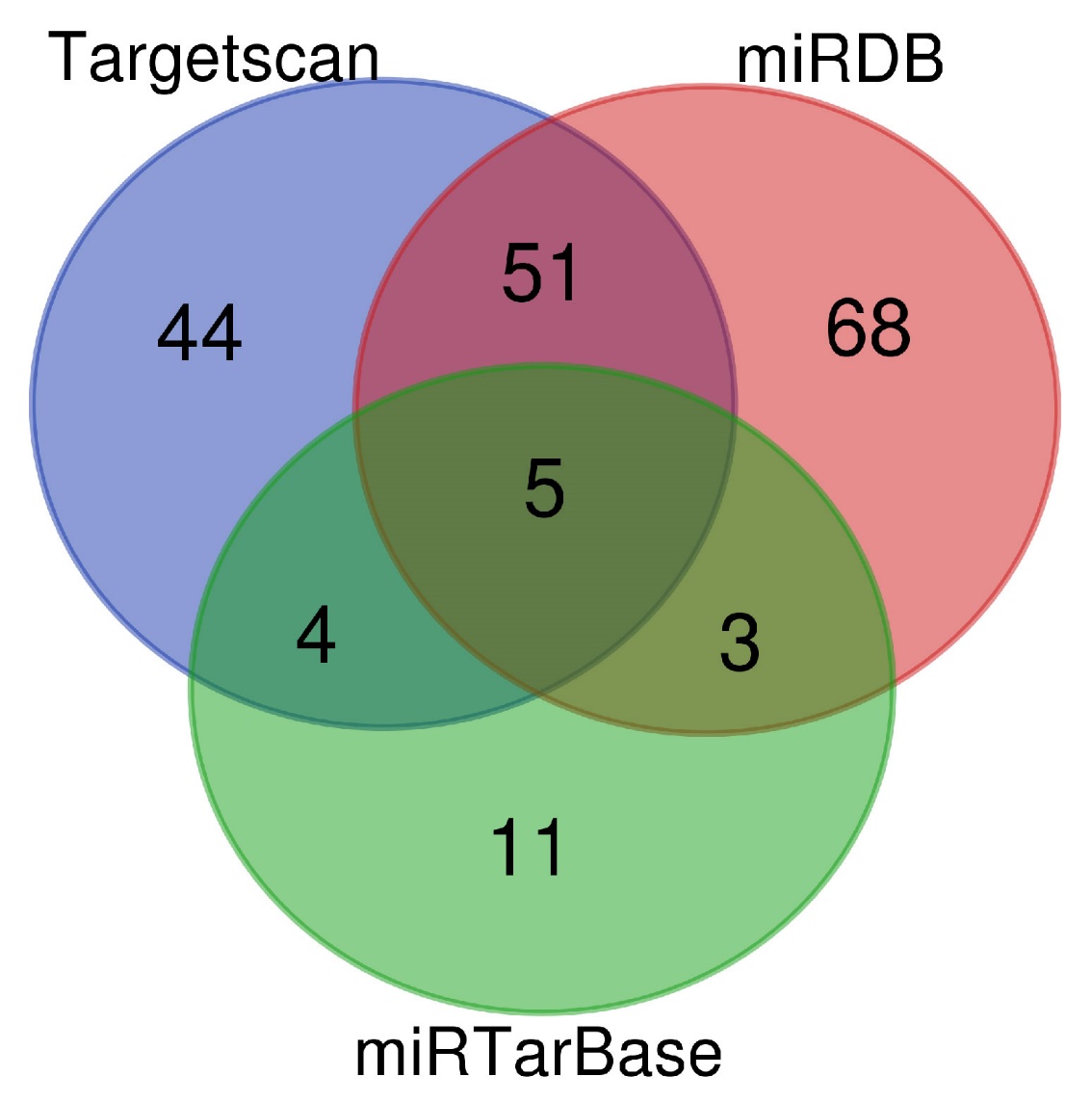


**Figure.S6.** **The Venn diagrams of main target gene of miR-139-5p**. The result showed that PDE4D, LCOR, ZBTB34, NR5A2, and DCBLD2 were the main target genes of miR-139-5p.


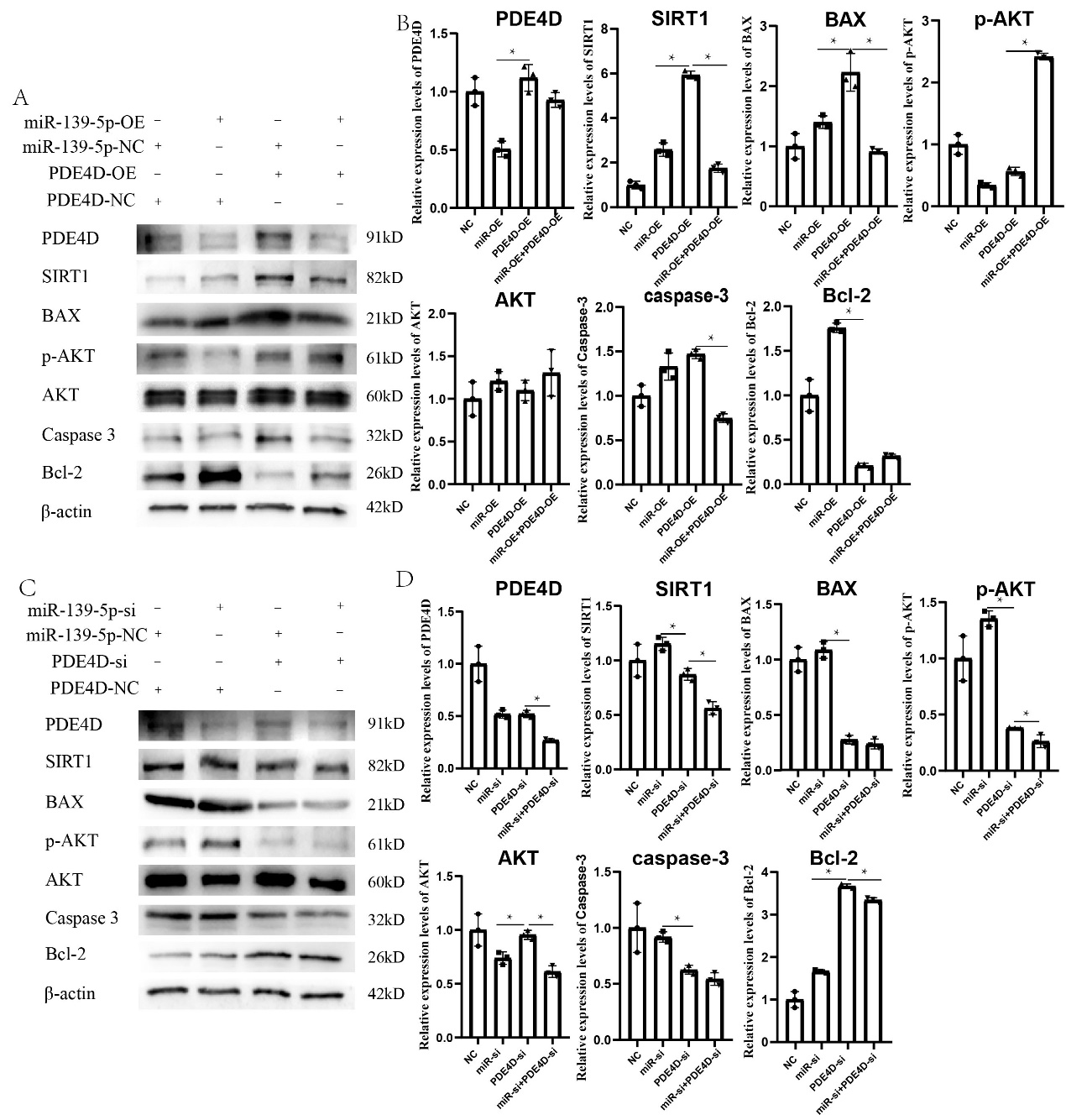


**Figure S7.** **Western blotting of PDE4D, SIRT1, AKT, p-AKT, Bcl-2, Bax, and Caspase 3 in HepaRG cell ALF model.** A: Western blotting of PDE4D, SIRT1, AKT, p-AKT, Bcl-2, Bax, and Caspase 3 in HepaRG cell ALF model transfected with miR-139-5p-OE, PDE4D-OE, miR-139-5p-OE+PDE4D-OE, or NC; B: Relative protein levels of PDE4D, SIRT1, AKT, p-AKT, Bcl-2, Bax, and Caspase 3 in HepaRG cell ALF model transfected with miR-139-5p-OE, PDE4D-OE, miR-139-5p-OE+PDE4D-OE, or NC; C: Western blotting of PDE4D, SIRT1, AKT, p-AKT, Bcl-2, Bax, and Caspase 3 in HepaRG cell ALF model transfected with miR-139-5p-si, PDE4D-si, miR-139-5p-si+PDE4D-si, or NC; D: Relative protein levels of PDE4D, SIRT1, AKT, p-AKT, Bcl-2, Bax, and Caspase 3 in HepaRG cell ALF model transfected with miR-139-5p-si, PDE4D-si, miR-139-5p-si+PDE4D-si, or NC. OE: Overexpression; si: silence.

**Table S1.** Antibodies used in flow cytometry, western blotting, and Ki 67 staining

| **Antibody** | **Supplier** | **Identifier** |
| --- | --- | --- |
| anti-human CD45 | Biolegend | 304017 |
| anti-human CD73 | Biolegend | 344004 |
| anti-human HLA-DR | Biolegend | 307616 |
| anti-human CD90 | Biolegend | 328114 |
| anti-human CD105 | Abcam | ab187575 |
| anti-human CD11b | Biolegend | 301306 |
| anti-human CD19 | Biolegend | 302216 |
| anti-human CD34 | Biolegend | 343608 |
| PE/Cy7 Mouse IgG2a, κIsotype Ctrl | Biolegend | 400232 |
| PE/Cy7 Mouse IgG2a, κIsotype Ctrl | Biolegend | 400126 |
| PE Mouse IgG1，κIsotype Ctrl | Biolegend | 400112 |
| APC Mouse IgG1，κIsotype Ctrl | Biolegend | 400122 |
| FITC Mouse IgG1，κIsotype Ctrl | Biolegend | 400129 |
| CD63 | Abcam | ab59479 |
| CD9 | Abcam | ab236630 |
| CD81 | Abcam | ab79559 |
| Syntenin | Abcam | ab133267 |
| TSG101 | Proteintech | 28283-1-AP |
| Calnexin | Proteintech | 10427-2-AP |
| Ki-67 | Fitzgerald | 10R-KR001 |
| PDE4D | Proteintech | 67062-1-lg |
| SIRT1 | Proteintech | 60303-1-lg |
| p-AKT | Proteintech | 66444-1-lg |
| Bcl-2 | Proteintech | 26593-1-AP |
| Bax | Proteintech | 60267-1-lg |
| Cleaved-caspase 3 | Affinity | AF7022 |

**Table S2**. hUCMSC-BAL rescue porcine ALF

| Group | Weight | Cell number | Treat Duration | Peak AST | Peak TBIL | Peak Amm | Survival time |
| --- | --- | --- | --- | --- | --- | --- | --- |
| A | 24 | 0 | 0 h | 4300 | 127 | 336 | 132 |
| A | 24 | 0 | 0 h | 3260 | 110 | 368 | 93 |
| A | 26 | 0 | 0 h | 5450 | 111 | 291 | 84 |
| A | 23.5 | 0 | 0 h | 7370 | 148 | 304 | 118 |
| A | 26 | 0 | 0 h | 8410 | 126 | 351 | 108 |
| B | 26 | 2.1x10^9^ | 8 h | 4860 | 115 | 384 | 108 |
| B | 26 | 2.3x10^9^ | 8 h | 1990 | 128 | 384 | 80 |
| B | 30 | 3.0x10^9^ | 8 h | 3350 | 94 | 132 | Survival |
| B | 26.5 | 2.2x10^9^ | 8 h | 2880 | 65 | 171 | 60 |
| B | 24 | 3.2x10^9^ | 8 h | 3130 | 57 | 113 | Survival |
| C | 25.5 | 1.4x10^10^ | 8 h | 1470 | 64 | 88 | Survival |
| C | 23 | 1.0x10^10^ | 8 h | 1630 | 93 | 72 | Survival |
| C | 26 | 1.2x10^10^ | 8 h | 272 | 13 | 84 | Survival |
| C | 23 | 2.1x10^10^ | 8 h | 489 | 7 | 60 | Survival |
| C | 27 | 1.8x10^10^ | 8 h | 1770 | 93 | 63 | Survival |
| Group A：ALF+ST; Group B: ALF+ST+low dose hUCMSC-BAL；Group C：ALF+ST+high dose hUCMSC-BAL | | | | | | | |

**Table S3.** Inflammatory factor detection by ELISA Kit

| **Name** | **Supplier** | **Identifier** |
| --- | --- | --- |
| IL-1 beta Porcine ELISA Kit | Invitrogen | Catalog # ESIL1B |
| Porcine TNF-alpha ELISA Kit | Invitrogen | Catalog # ES24RB |
| IL-18 Porcine ELISA Kit | Invitrogen | Catalog # BMS672 |
| IL-6 Porcine ELISA Kit | Invitrogen | Catalog # ESIL6 |
| Porcine IL-8 ELISA Kit | Invitrogen | Catalog # ES15RB |
| IL-10 Porcine ELISA Kit | Invitrogen | Catalog # KSC0101 |
| IL-12 Porcine ELISA Kit | Invitrogen | Catalog # ESIL12A |
| Porcine IL-1alpha ELISA Kit | Invitrogen | Catalog # ES10RB |
| Porcine IFN-gamma ELISA Kit | Invitrogen | Catalog # ES9RB |
| Porcine VEGF-A ELISA Kit | Invitrogen | Catalog # ES25RB |
| Porcine HGF ELISA Kit | Dogesce | DG50140P-48T |

**Table S4.** Primers for real-time PCR

| **Gene** | **Forward (5’-3’)** | **Reverse (5’-3’)** |
| --- | --- | --- |
| miR-139-5p | ACACTCCAGCTGCACGTGTC | TGGTGTCGTGGAGTCGGTTGA |
| U6 | CTCGCTTCGGCAGCACA | AACGCTTCACGAATTTGCGT |
| PDE4D | TTTTGCCAGTGCAATACATGATG | CAGAGCGAGTTCCGAGTTTGT |
| SIRT1 | GCTGACGACTTCGACGACG | TCGGTCAACAGGAGGTTGTCT |
| Bcl-2 | ATAACGGAGGCTGGGTAGGT | CCAGAATCCACTCACACCCC |
| Bax | TGAAGACAGGGGCCTTTTTG | AATTCGCCGGAGACACTCG |
| Caspase 3 | ATGGAGAACAACAAAACCTCAGT | TTGCTCCCATGTATGGTCTTTAC |
